# N6-methyladenosine-modification of USP15 regulates chemotherapy resistance by inhibiting LGALS3 ubiquitin-mediated degradation via AKT/mTOR signaling activation pathway in hepatocellular carcinoma

**DOI:** 10.1038/s41420-024-02282-y

**Published:** 2025-01-10

**Authors:** Ronghuan Fang, Zhigang Jia, Yuhang Xin, Kai Zhao, Wei Qin, Haoran Lu, Yi Zhou, Yongsheng Yang, He Fang

**Affiliations:** 1 Department of Hepatobiliary Pancreatic Surgery, The Second Hospital of Jilin University, Changchun, China; 2 Jilin Engineering Laboratory for Translational Medicine of Hepatobiliary and Pancreatic Diseases, Changchun, China; 3 Department of Hepatobiliary Surgery, Afliated Hospital of Jining Medical, Jining, China; 4 Guangxi Key Laboratory of Early Prevention and Treatment for Regional High Frequency Tumor, Nanning, China

**Keywords:** Hepatocellular carcinoma, Hepatocellular carcinoma, Mechanisms of disease, Epigenomics, Ubiquitylation

## Abstract

Hepatocellular carcinoma (HCC) is among the most malignant tumors and seriously threatens human health worldwide, and its incidence rate is increasing annually. USP15 is a member of the ubiquitination-specific protease (USP) family, which can regulate protein ubiquitination, thereby affecting their stability, and is dysregulated in many cancers, but its expression and regulatory mechanism in HCC are unclear. The aims of this study were to explore the role and mechanism of USP15 in regulating HCC cell stemness, proliferation, and lenvatinib resistance. Immunohistochemistry and high-throughput sequencing analyses of tumor and adjacent normal tissue samples from 52 patients with HCC were conducted. Functional analyses of immortalized human liver and HCC cell lines were conducted, including quantitative real-time PCR; western blot; plasmid, lentivirus, and siRNA transfection; co-immunoprecipitation; mass spectrometry; MeRIP-qPCR; and ubiquitination, cell growth, colony formation, and spheroid formation assays. HCC tumor growth was also assessed using cell transplantation in nude mice. We found that USP15 is upregulated in HCC and affects patient prognosis. Our results demonstrated that USP15 can increase LGALS3 stability in HCC through deubiquitination modification, and affect the stemness, proliferation, and lenvatinib resistance of HCC cells by activating the AKT/mTOR pathway. USP15 expression levels were positively correlated with HCC cell stemness, proliferation, and lenvatinib resistance. In addition, methyltransferase-like protein 3 (Mettl3) N6-methyladenosine (m6A) modified USP15 to upregulate its levels by increasing its mRNA stability. These findings provide a theoretical basis for the potential discovery of new HCC oncogenes, as well as the identification of effective targets and development of novel anti-HCC drugs and clinical applications.

## Background

HCC is one of the most common malignant tumors and its incidence continues to increase worldwide. HCC is extremely malignant, with a 5-year overall survival rate (OS) of just 18%. Its onset is insidious, and by the time of diagnosis, approximately 80% of HCC patients have already reached an advanced stage, at which point surgery is no longer feasible [1, 2].

Ubiquitination is a reversible post-translational modification that regulates protein degradation and signal transduction by covalently binding small molecules of ubiquitin to target proteins. The process of ubiquitination leads to protein degradation through ubiquitin-proteasomes and selective autophagy [3], and is involved in many cellular life activities, such as cell cycle regulation, DNA repair, and signal transduction. Ubiquitination is also involved in regulating tumor cell proliferation, metastasis, and angiogenesis [4]. Deubiquitinases can regulate the ubiquitination modification of proteins, thereby affecting their stability. The USP family, which includes USP15, constitutes the largest subfamily of deubiquitinases.

USP15 is involved in various cell biological processes, such as proliferation, invasion, apoptosis, and transcriptional regulation, among others. Further, USP15 is upregulated in several malignancies including breast, ovarian, gastric, and bladder cancers, but downregulated in around 25% of pancreatic cancer and 11% of glioblastoma, indicating that USP15 potentially has dual roles in promoting and suppressing tumors [5–10].

It has been reported in that USP15 is highly expressed in gastric cancer, promoting malignant progression by activating the Wnt/β-catenin pathway [8], however, other studies have found that USP15 can inhibit the NF-κB pathway by deubiquitinating IκB-α, thereby inhibiting the proliferation and invasion of gastric cancer [11]. In the context of HCC, some studies have shown that USP15 is highly expressed in tumor tissues, promoting HCC cell proliferation and inhibiting apoptosis [12]. However, another investigation found that USP15 increases KEAP1 stability through deubiquitination, thereby promoting Nrf2 degradation, which results in reactive oxygen species accumulation, ultimately inducing HCC cell apoptosis and inhibiting HCC proliferation [13]. These studies demonstrate that the biological function of USP15 in cancers is complex and even contradictory. Hence, in-depth understanding of the role and mechanism underlying USP15 activity in HCC will be conducive to determining the processes involved in HCC occurrence and development, which are of great significance for the treatment of this type of cancer.

In this study, we found that USP15 is highly expressed in HCC and affects patient prognosis. Further, our data show that USP15 can increase LGALS3 stability through deubiquitination modification, promote HCC cell stemness and proliferation by activating the AKT/mTOR pathway, and improve HCC resistance to lenvatinib. In addition, our results indicate that m6A modification by Mettl3 maintains high USP15 expression by increasing USP15 mRNA stability.

## Results

### Expression characteristics and clinical significance of USP15 in HCC

**Fig. 1:**
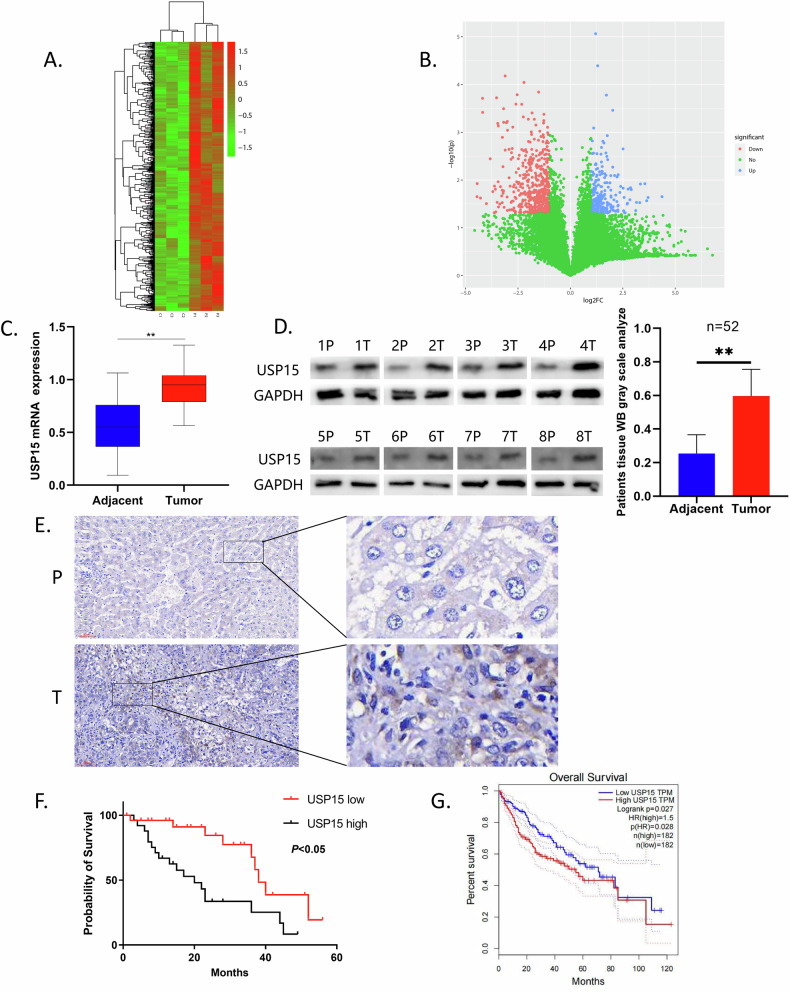
Expression characteristics and clinical significance of USP15 in HCC. **A**, **B** Heat maps and volcano plots of the results of high-throughput sequencing of patient tissue specimens showing the difference in *USP15* expression between tumor and adjacent normal tissues(**A** Green represents tumor tissues, and red represents para - cancerous tissues; **B** Blue indicates high - expression, and red indicates low - expression). **C**, **D** Comparison of USP15 expression in tumor and paired adjacent normal tissues from 52 patients with HCC detected by qRT-PCR and WB (52 specimens were examined, since 8 of them are the most representative and sufficient to stand for all 52 specimens, we did not insert all the specimens into the figure). **E** Comparison of USP15 expression in tumor and paired normal adjacent tissues from patients with HCC detected by immunohistochemistry(Detected at a magnification of 200 times with a scale bar of 60μm). **F** Kaplan-Meier analysis of the relationship between USP15 expression levels and prognosis in 52 patients with HCC. **G** Analysis of the relationship between USP15 expression and prognosis of patients with HCC using data from the GEPIA database. Data are expressed as mean values, *p < 0.05, **p < 0.01.

We collected specimens from 52 patients with HCC who were treated at the Second Hospital of Jilin University from January 2019 to January 2021. Through high-throughput sequencing, we found that the *USP15* expression was higher in cancer tissue than that in paired adjacent tissues, suggesting that USP15 may affect HCC malignant progression (Fig. 1A, B). Subsequently, we performed qRT-PCR, WB, and immunohistochemistry analyses of the specimens from the 52 patients with HCC finding that USP15 expression levels in cancer tissues were significantly higher than those in adjacent tissues (Fig. 1C–E). In addition, analysis of patient data indicated that USP15 expression level was positively correlated with tumor TNM stage, tumor size, cirrhosis, and microvascular invasion, while negatively correlated with patient overall survival (Supplementary Table S2; Fig. 1F). Data analysis from the GEPIA database (GEPIA (cancer-pku.cn))also showed that USP15 expression level was negatively associated with both overall survival and disease-free survival in patients with HCC (Fig. 1G; Supplementary Fig. S1C).

### Effect of USP15 on HCC cell stemness

**Fig. 2:**
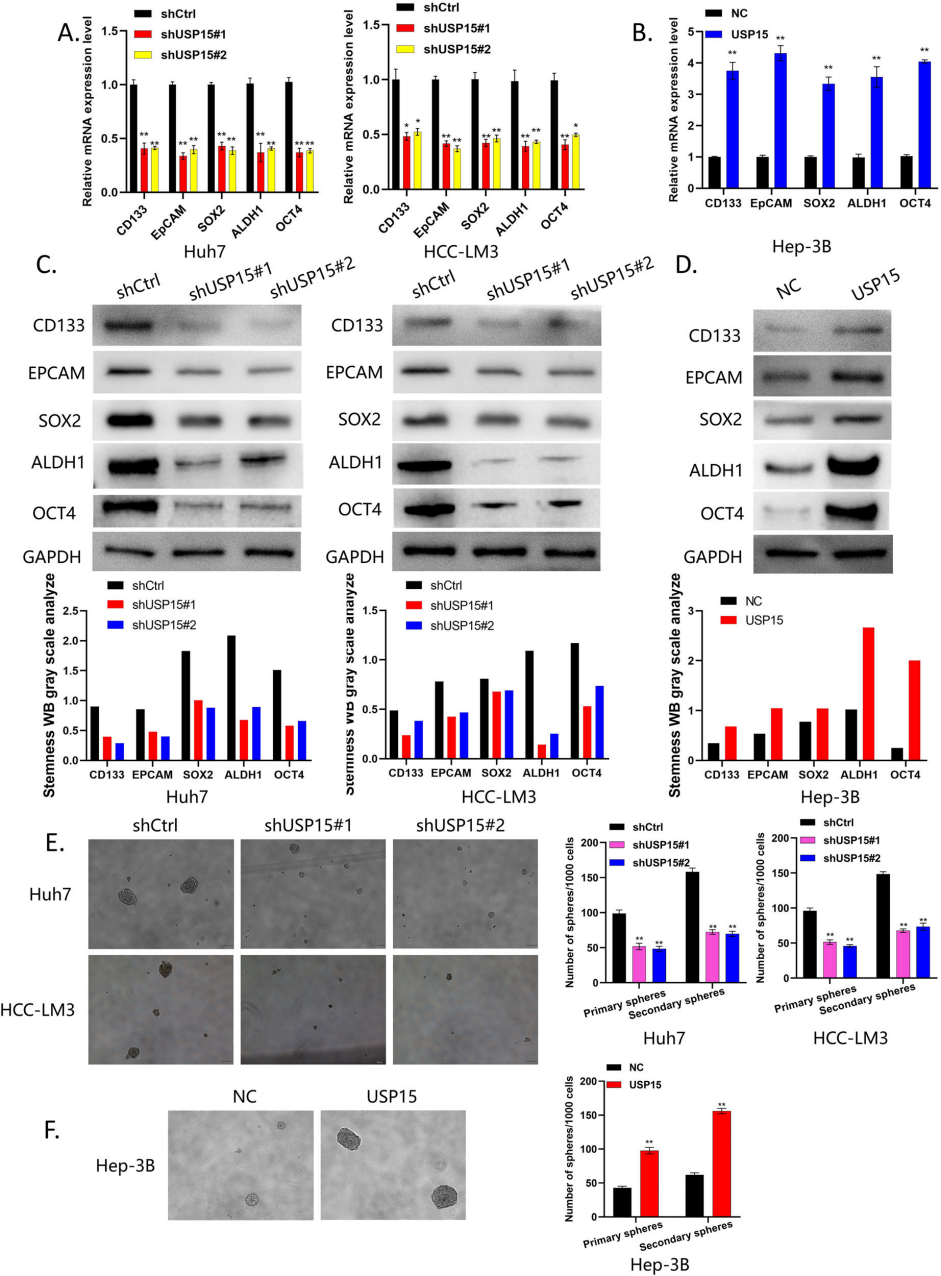
Effect of USP15 on HCC cell stemness. **A,** **B** qRT-PCR analysis of expression of the tumor stemness-related genes, *CD133*, *EPCAM*, *SOX2*, *ALDH1* and *OCT4*, in Huh7 and HCC-LM3 cells with USP15 knocked down and Hep3B cells overexpressing USP15. **C,** **D** WB analysis of expression of the tumor stemness-related proteins, CD133, EPCAM, SOX2, ALDH1 and OCT4 in Huh7 and HCC-LM3 cells with USP15 knocked down and Hep3B cells overexpressing USP15. **E,** **F** Sphere-formation assay to assess the primary and secondary sphere formation abilities of Huh7 and HCC-LM3 cells with USP15 knocked down and Hep3B cells overexpressing USP15. Images show secondary spheres formed by these cells. Data are expressed as mean values, *p < 0.05, **p < 0.01.

Our data suggest that USP15 may promote HCC malignant progression, though the specific function of USP15 in HCC remains unknown. First, the experimental cell lines Huh-7 (high USP15 expression), HCC-LM3 (high USP15 expression) and Hep-3B (relatively low USP15 expression) were screened through qRT-PCR and WB experiments, and stable knockdown of USP15 conducted by lentiviral transfection. The resulting cell lines were the USP15-knockdown Huh-7, HCC-LM3, and the USP15-overexpressing cell line, Hep-3B (Supplementary Figs. S1A,B; S2A, B). Next, we conducted qRT-PCR and WB experiments, which demonstrated positive correlations between levels of USP15 and those of molecules related to tumor stemness (CD133, EPCAM, SOX2, ALDH1 and OCT4) [14, 15] (Fig. 2A–D). Further, in sphere-forming experiments, the knockdown of USP15 significantly reduced the sphere-forming ability of Huh-7 and HCC-LM3 cells, while that of Hep-3B cells overexpressing USP15 was enhanced (Fig. 2E, F), indicating that USP15 promotes HCC cell stemness.

### Effect of USP15 on HCC proliferation and lenvatinib resistance

**Fig. 3:**
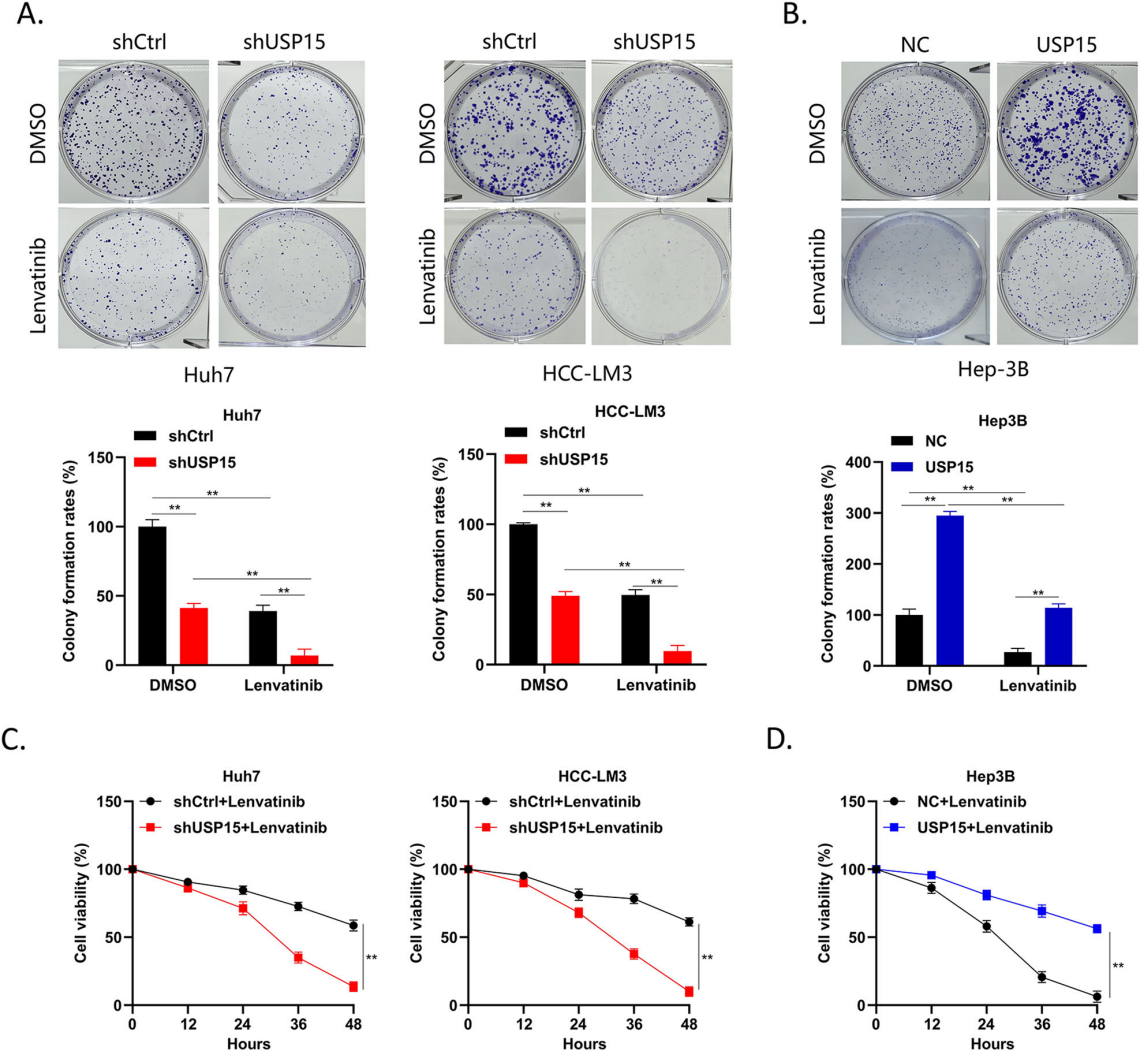
Effects of USP15 on HCC cell proliferation and lenvatinib resistance. **A,** **B** Colony formation assay to analyze the colony formation rate of Huh7 and HCC-LM3 cells with USP15 knocked down (**A**) and Hep3B cells overexpressing USP15 (**B**) following treatment with lenvatinib. **C,** **D** CCK8 assay to analyze the survival rate of Huh7 and HCC-LM3 cells with USP15 knocked down (**C**) and Hep3B cells overexpressing USP15 (**D**) following treatment with lenvatinib. Data are expressed as mean values, *p < 0.05, **p < 0.01.

Several studies have shown that tumor drug resistance is significantly related to their stemness properties [16, 17]. Therefore, we examined whether USP15 influences HCC resistance to lenvatinib. In colony formation experiments, the same concentration of lenvatinib was added to each experimental group, and the colony formation rate of Huh-7 and HCC-LM3 cells with USP15 knocked down was significantly lower than that of corresponding wild-type HCC cells, DMSO was added to cells with USP15 knocked down as a control group. The colony formation rates of Huh-7 and HCC-LM3 cells were also significantly lower than those of wild-type HCC cells. In Hep-3B overexpressing USP15, the opposite results were observed (Fig. 3A, B). Additionally, a CCK8 experiment conducted by us showed that, over time, the viability of USP15-knockdown Huh-7 and HCC-LM3 cells treated with the same concentration of lenvatinib was lower than that of wild-type HCC cells. Conversely, in USP15-overexpressing Hep-3B cells, the opposite results were observed (Fig. 3C, D). These findings demonstrate that USP15 promotes both HCC cell proliferation and increases resistance to lenvatinib in HCC cells.

### In vivo experiments

**Fig. 4:**
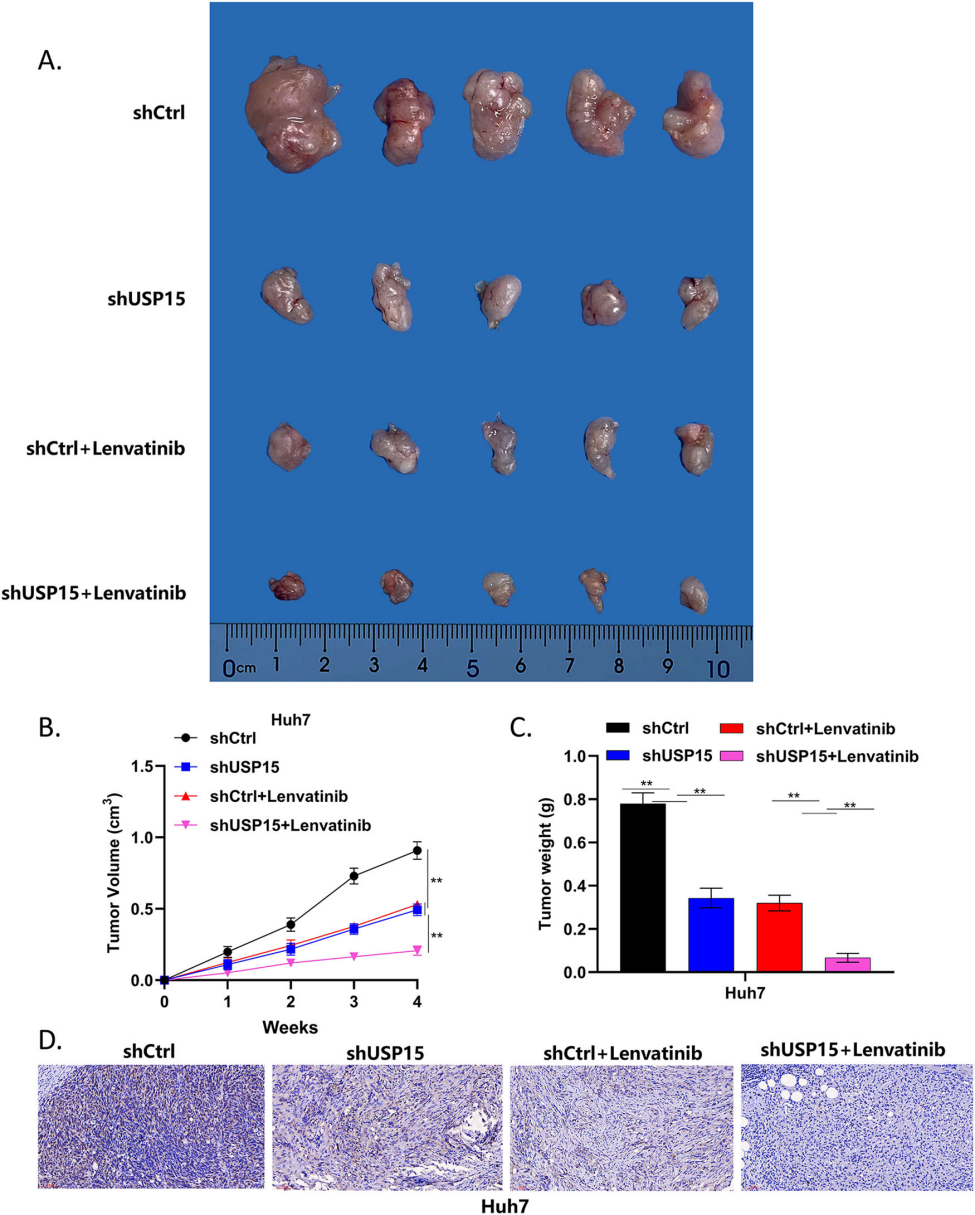
USP15 promotes HCC proliferation and improves lenvatinib resistance in vivo. **A** Nude mouse tumor formation experiment. Compared with mice injected with wild-type Huh7 cells, those inoculated with Huh7 cells with USP15 knocked down generated significantly smaller subcutaneous tumors. After injection of mice with lenvatinib via the tail vein, mice inoculated with Huh7 cells with USP15 knocked down had smaller subcutaneous tumors than those in mice inoculated with wild-type Huh7 cells. **B** Subcutaneous transplanted tumor volume in nude mice of each group. **C** Subcutaneous transplanted tumor mass in each group of nude mice. **D** Immunohistochemical analysis of the expression of USP15 in subcutaneous transplanted tumor specimens of four groups of nude mice (Detected at a magnification of 200 times with a scale bar of 60μm). Data are expressed as mean values, *p < 0.05, **p < 0.01.

To further verify the above results, we conducted a tumor formation experiment in nude mice. Tumors transplanted in nude mice inoculated with Huh7-shUSP15 were significantly smaller than those in mice inoculated with wild-type Huh7 cells. Additionally, when nude mice in both the experimental and control groups received 50 mg/kg lenvatinib injections via the tail vein, the transplanted tumors in mice inoculated with Huh7-shUSP15 cells were smaller than those in mice inoculated with wild-type Huh7 cells (Fig. 4A–C). Immunohistochemistry analysis of tumor tissue samples from mice in each group was also conducted to assess USP15 expression levels (Fig. 4D).

Overall, our data indicate that USP15 is highly expressed in HCC and is negatively correlated with patient prognosis. Our findings also indicate that USP15 promotes HCC cell stemness and proliferation, as well as enhancing resistance to lenvatinib in HCC.

### LGALS3 is downstream of USP15 and LGALS3 interacts with USP15 to promote HCC stemness, proliferation, and lenvatinib resistance through AKT/m-TOR activation

**Fig. 5:**
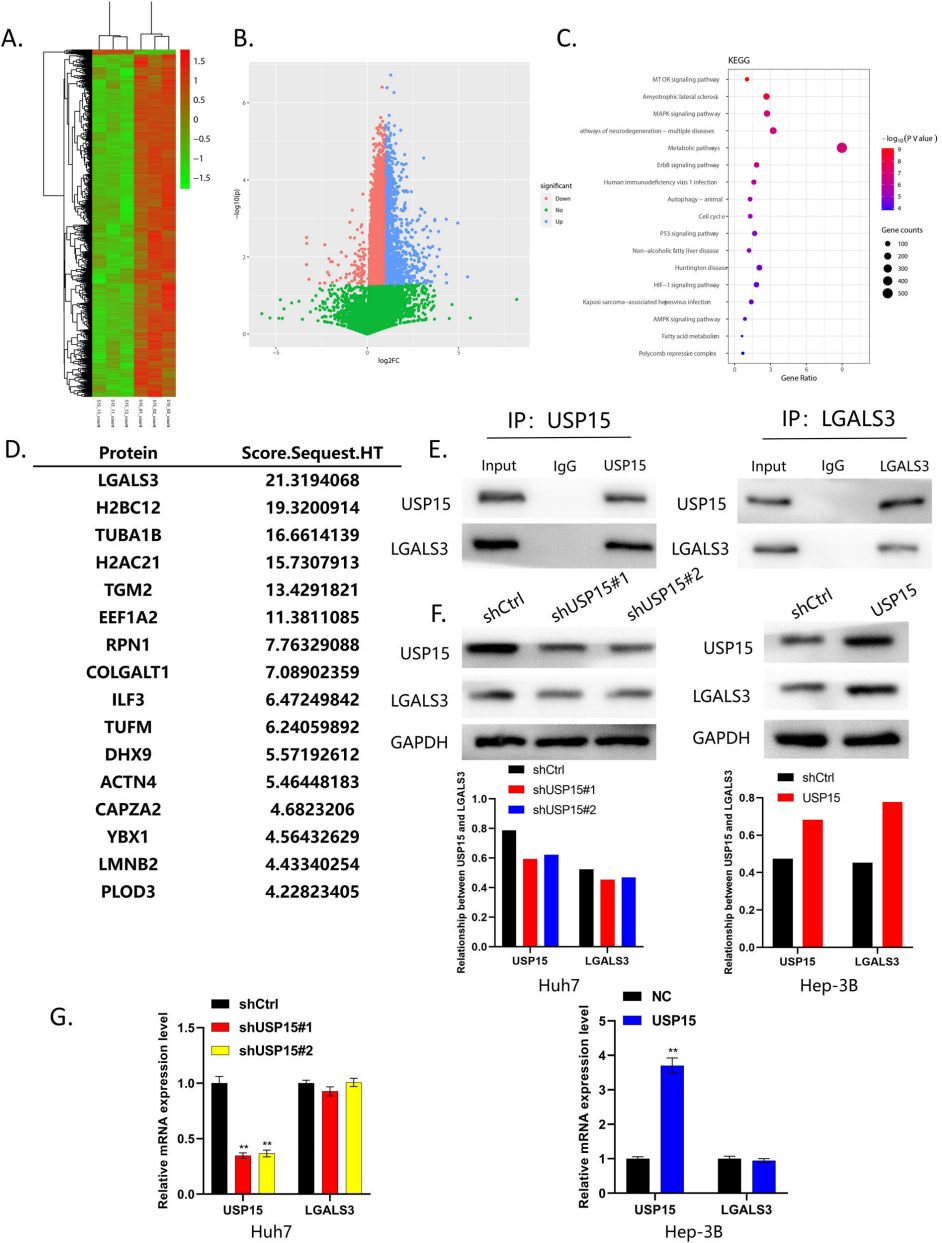
LGALS3 is located downstream of USP15 and its protein expression is regulated by USP15. **A, B** High-throughput sequencing analysis of HCC cells differentially expressing USP15; heat maps and volcano maps showing differentially expressed genes (Blue indicates high - expression, and red indicates low - expression). **C** KEGG analysis of pathways enriched for differentially expressed genes. **D** Mass spectrometry analysis identifying LGALS3 as a molecule co-expressed with USP15. **E** Immunoprecipitation experiments verifying that USP15 and LGALS3 can physically interact. **F, G** WB and qRT-PCR experiments to analyze the relationship between USP15 and LGALS3. USP15 and LGALS3 were positively correlated at the protein, but not at the mRNA, level. Data are expressed as mean values, *p < 0.05, **p < 0.01.

In previous experiments, we found that USP15 can promote HCC cell stemness and proliferation and increase HCC resistance to lenvatinib, but the specific mechanism involved remains to be elucidated. Therefore, we conducted high-throughput sequencing of samples from HCC cells that expressed different levels of USP15 to identify differentially expressed genes, followed by KEGG pathway enrichment analysis. The results indicated that USP15 may act through the AKT/m-TOR pathway (Fig. 5A–C).

To explore proteins that interact with USP15, we next performed mass spectrometry analysis on transfected HCC cells and identified LGALS3 as a molecule co-expressed with USP15. We then performed Co-IP experiments and found that USP15 and LGALS3 could physically interact (Fig. 5D, E). To further explore the relationship between these two molecules, we performed qRT-PCR and WB experiments finding that LGALS3 is downstream of USP15. Additionally, our data indicate that USP15 can regulate LGALS3 at the protein level, while knockdown or overexpression of *USP15* at the mRNA level does not affect on LGALS3 (Fig. 5F, G).

**Fig. 6:**
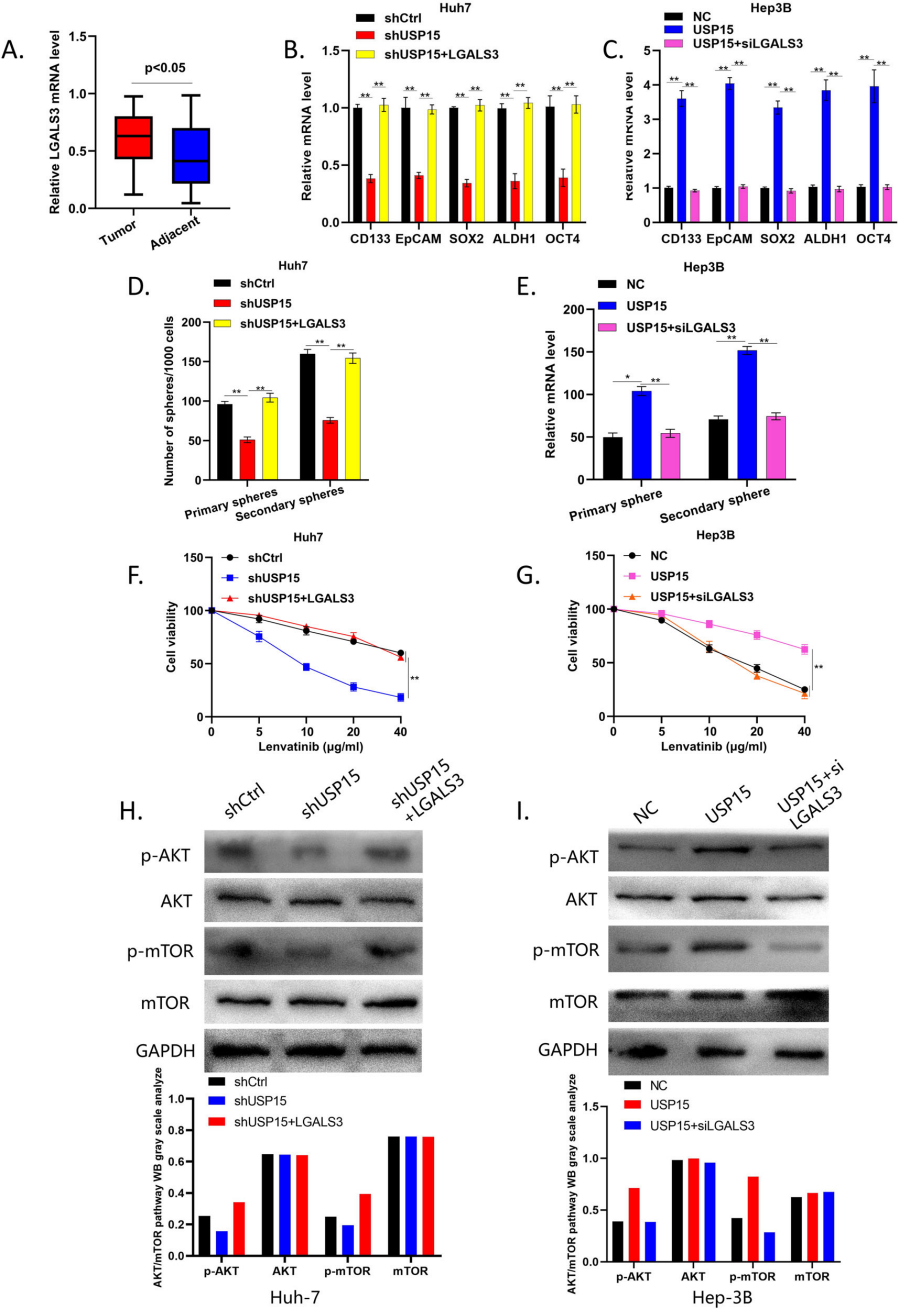
LGALS3 interacts with USP15 to promote HCC stemness, proliferation, and lenvatinib resistance through AKT/m-TOR activation. **A** qRT-PCR analysis of *LGALS3* expression in tumor and paired adjacent tissues from 52 patients with HCC. **B, C** qRT-PCR analysis of the expression of the tumor stemness-related genes *CD133*, *EPCAM*, *SOX2*, *ALDH1* and *OCT4* in Huh7 cells with USP15 knocked down and LGALS3 overexpressed, and in Hep3B cells with USP15 overexpressed and LGALS3 knocked down. **D,** **E** Analysis of the primary and secondary sphere formation abilities of Huh7 cells with USP15 knocked down and LGALS3 overexpressed, and of Hep3B cells with USP15 overexpressed and LGALS3 knocked down. **F,** **G** CCK8 assay to analyze survival rates following treatment with lenvatinib of Huh7 cells with USP15 knocked down and LGALS3 overexpressed and of Hep3B cells with USP15 overexpressed and LGALS3 knocked down. **H,** **I** WB assay of AKT/m-TOR pathway-related protein expression in Huh7 and Hep3B cells differentially expressing USP15 and LGALS3. Data are expressed as mean values, *p < 0.05, **p < 0.01.

LGALS3, a member of the galectin family, has been reported to be closely associated with tumor occurrence and development [18]. To investigate the biological role of LGALS3 in HCC, we repeated our previous experiments by performing qRT-PCR on 52 HCC tissue specimens. We found that LGALS3 was highly expressed in HCC tissues (Fig. 6A), indicating that it may play an oncogenic role. In subsequent experiments, we used siRNA and transfection to construct lines with LGALS3 overexpressed and knocked down in Huh-7 and Hep-3B cells stably transfected with USP15. We further performed qRT-PCR, spheroid formation, and CCK8 assays on these cell lines and found that, similar to USP15, LGALS3 promotes HCC cell stemness and proliferation and enhances their resistance to lenvatinib (Fig. 6B–G).

Therefore, we hypothesize that USP15 and LGALS3 act together to influence HCC malignant progression, though the pathways through which they operate remain to be verified. We subsequently performed WB on HCC cell lines with differential USP15 and LGALS3 expression to assess the levels of proteins associated with the AKT/mTOR pathway. The results showed that USP15 and LGALS3 act together to increase the expression levels of p-AKT and p-mTOR. This activation of the AKT/mTOR pathway promotes HCC cell stemness and proliferation and enhances resistance to lenvatinib (Fig. 6H, I).

### USP15 mediates LGALS3 expression through deubiquitination modification

**Fig. 7:**
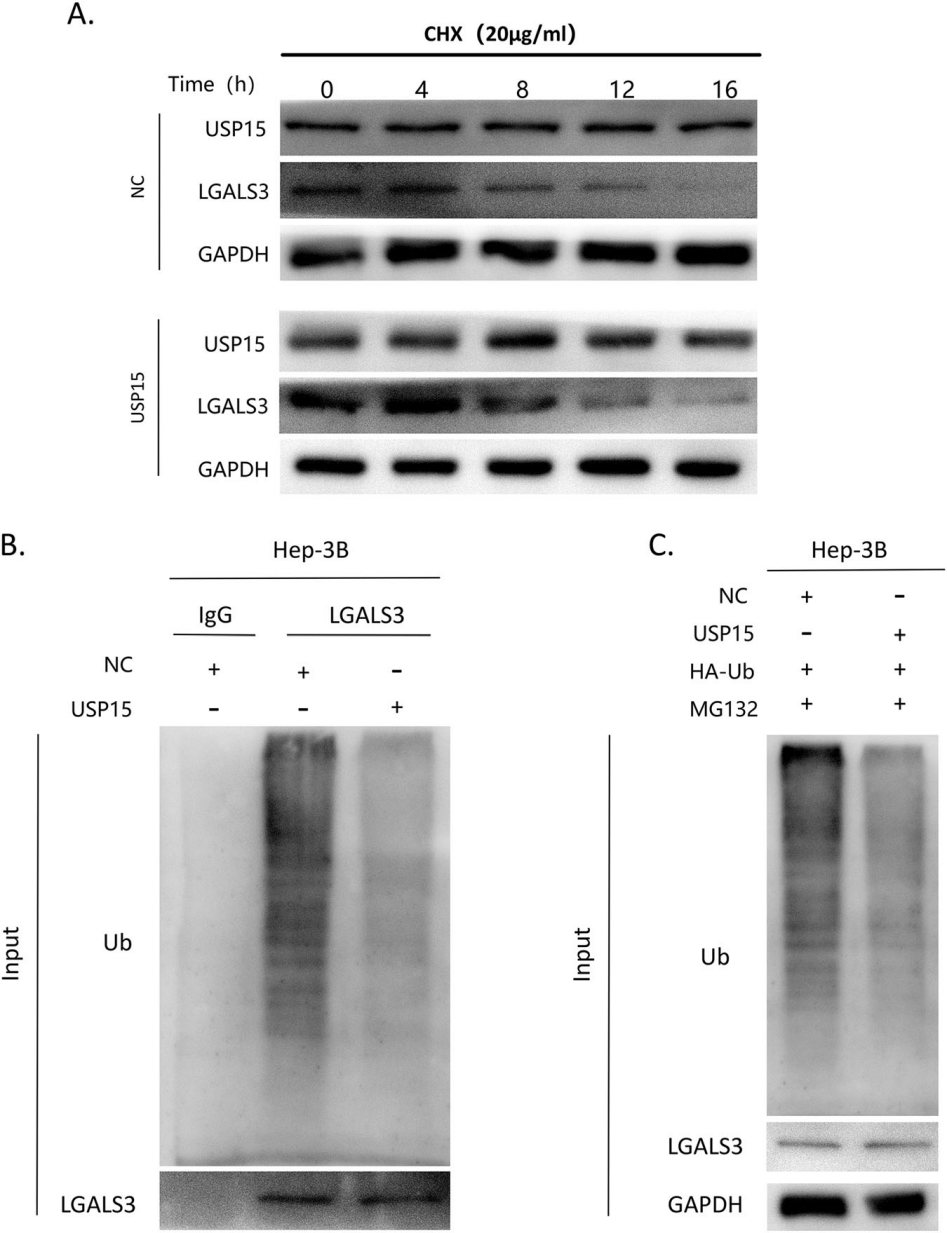
USP15 mediates LGALS3 expression through deubiquitination modification. **A** Protein half-life assay to assess LGALS3 stability in wild-type and USP15-overexpressing Hep3B cells. LGALS3 protein stability was increased in Hep3B cells overexpressing USP15. **B,** **C** Ubiquitination assay to analyze the effect of USP15 on LGALS3 ubiquitination-mediated degradation. LGALS3 ubiquitination-mediated degradation was significantly reduced following USP15 overexpression.

In previous experiments, we found that USP15 and LGALS3 can interact, and that both are up-regulated in HCC. To explore whether there is a correlation between the high expression levels of USP15 and LGALS3, we conducted a protein half-life experiment. The results showed that overexpression of USP15 significantly increased the stability of LGALS3 (Fig. 7A). Given that USP15 is a deubiquitinating enzyme, we hypothesized that USP15 may improve LGALS3 stability by inhibiting its ubiquitination and degradation. To test this hypothesis, we conducted ubiquitination experiments and found that USP15 overexpression inhibited the LGALS3 ubiquitination-mediated degradation, proving that USP15 affects LGALS3 stability through deubiquitination modification (Fig. 7B, C). This finding also clarifies our data showing that USP15 regulates LGALS3 at the protein level without affecting its mRNA level.

### m6A modification mediates USP15 expression upregulation in HCC

Analysis using RMBase (https://rna.sysu.edu.cn/rmbase/m6Amod.php) indicated that *USP15* contains numerous m6A binding sites. Therefore, we hypothesize that high USP15 expression in HCC is related to its m6A modification, which is the most common and abundant post-transcriptional modification in eukaryotic RNA. Mettl3 is a methyltransferase that is key to regulation of m6A modification. Mettl3 expression is upregulated in HCC, but its specific functional mechanism in this context is unclear [19].

**Fig. 8:**
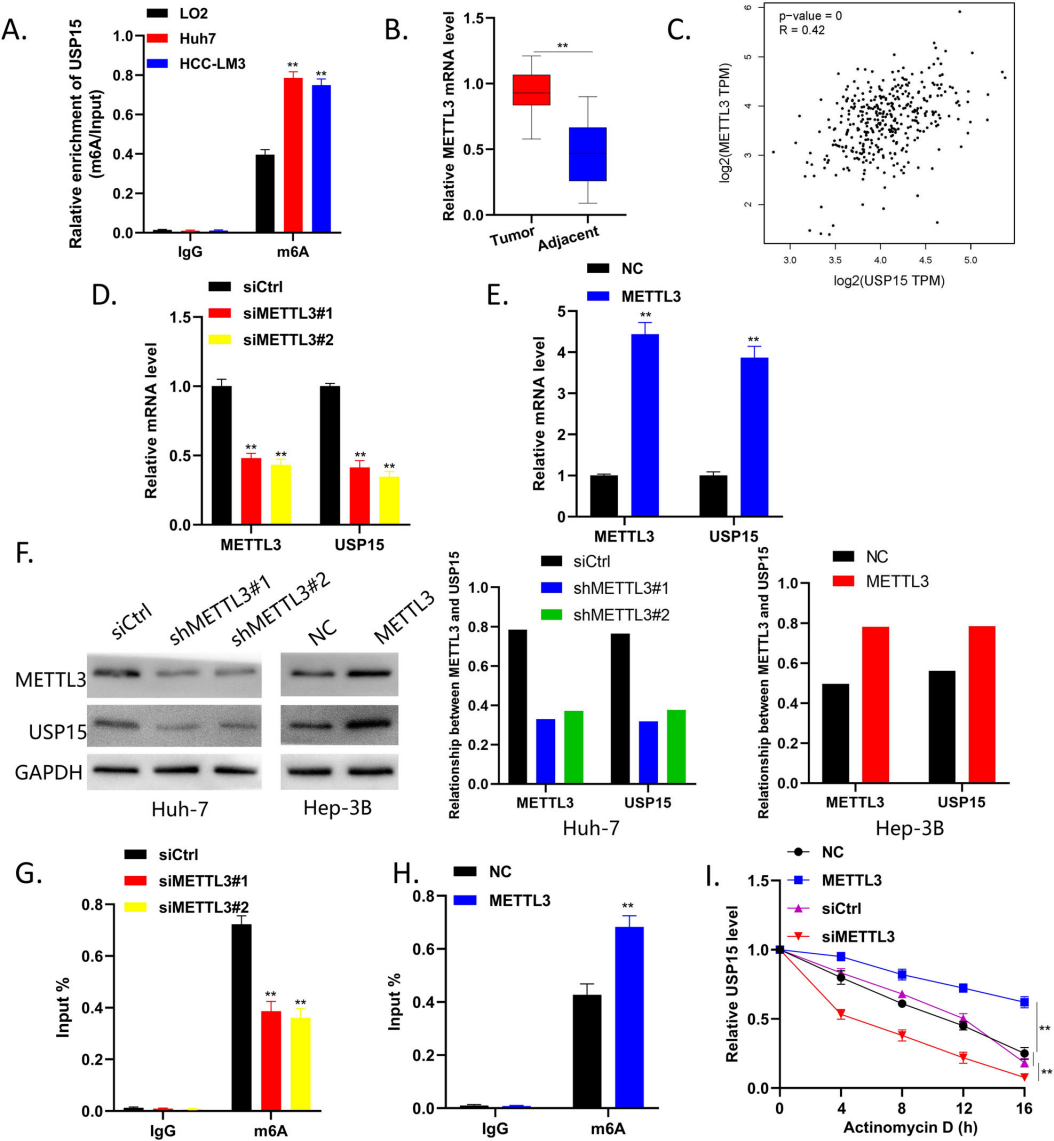
m6A modification mediates USP15 expression upregulation in HCC. **A** MeRIP-qPCR assay to detect m6A enrichment in Huh7 and HCC-LM3 cells. **B** qRT-PCR assay to detect *Mettl3* expression levels in tumor and paired adjacent normal tissues from 52 patients with HCC. **C** Analysis of data from the GEPIA database showing that Mettl3 expression level was positively correlated with that of USP15. **D,** **E** qRT-PCR analysis of the effect of Mettl3 knockdown or overexpression on *USP15* expression. **F** WB assay to analyze the effect of Mettl3 knockdown or overexpression on USP15 expression. **G**, **H** MeRIP-qPCR assay to analyze the effect of Mettl3 knockdown or overexpression on m6A enrichment in the *USP15* gene. **I** Actinomycin D assay to determine the effect of Mettl3 knockdown and overexpression on USP15 stability. Data are expressed as mean values, *p < 0.05, **p < 0.01.

First, we conducted MeRIP-qPCR experiments, which demonstrated significantly higher *USP15* m6A enrichment levels in the Huh-7 and HCC-LM3 cell lines than that in the immortalized human liver cell line, LO2. Next, we performed qRT-PCR analysis on samples from the 52 pairs of HCC tissue samples described above finding that *Mettl3* levels were significantly higher in HCC tumor specimens than those in para-cancerous tissues, analysis of GEPIA database data further verified this result (Fig. 8A–C).

We then used siRNA and transfection to construct HCC cell lines with Mettl3 overexpressed and knocked down. Through qRT-PCR and WB experiments, we found that overexpression of Mettl3 significantly increased levels of USP15, while knocking down Mettl3 reduced USP15 expression (Fig. 8D–F). MeRIP-qPCR experiments showed that m6A enrichment of *USP15* in HCC cells decreased after the knockdown of Mettl3, while Mettl3 overexpression significantly increased the m6A enrichment of *USP15*. In an actinomycin D experiment, knockdown of Mettl3 reduced USP15 stability, while overexpressing Mettl3 increased USP15 stability (Fig. 8G–I). These data indicate that m6A modification mediates the high USP15 expression observed in HCC.

## Discussion

USP15 is a deubiquitinase that can increase protein stability through deubiquitination modification and participate in various intracellular processes, such as cell proliferation, apoptosis, autophagy, and immune responses [20, 21]. Moreover, USP15 is upregulated in many cancers and increases the stability of oncoproteins through deubiquitination modification, thereby promoting cancer progression [22]. However, in some tumors, there is evidence that USP15 has both pro-oncogenic and tumor suppressor roles [11, 13], indicating that the role of USP15 in cancer is complex and sometimes contradictory, and highlighting it as an important and promising target in tumor treatment. With the incidence and mortality of HCC are increasing annually, understanding the role and mechanisms of USP15 activity in HCC is of great significance.

The mechanism underlying HCC drug resistance is highly complex. This resistance may be related to the biological function of the liver, which can metabolize drugs, making it difficult for drugs to reach therapeutic concentrations in this organ. Drug resistance of HCC may also be related to increased expression of transporters, inactivation of signaling pathways that regulate apoptosis, redistribution of drugs in cells, and activation of tumor stem cells [23]. In this study, we demonstrate the role of USP15 in HCC progression and lenvatinib resistance and, for the first time, confirm that Mettl3-mediated m6A modification is involved in USP15 upregulation in HCC. In summary, our findings demonstrate that USP15 is highly expressed in HCC, with levels positively correlated with serum AFP levels, tumor TNM stage, tumor size, cirrhosis, and microvascular invasion, and negatively correlated with patient overall survival. Both in vitro and in vivo experiments demonstrated that USP15 promotes HCC stemness, proliferation, and increases HCC resistance to lenvatinib.

LGALS3 is a member of the galectin family that is mainly located in the cell cytoplasm. The N-terminus of LGALS3 is sensitive to matrix metalloproteinases and can interact with some proteins to participate in cell proliferation, apoptosis, pre-mRNA splicing, angiogenesis, inflammation, and fibrosis [24]. LGALS3 is a stable biomarker with elevated expression levels in many diseases, including heart, kidney, and liver disorders, as well as cancer [25]. In tumors, LGALS3 is upregulated in glioblastoma and can increase its sensitivity to radiotherapy and chemotherapy [26]. In addition, LGALS3 plays significant roles in hepatitis, cirrhosis, and liver failure. It is also highly expressed in HCC, where it may have a pro-cancer function [27]. In this study, we found that LGALS3 functions downstream of USP15 and is upregulated in HCC, where it can interact with USP15 to promote HCC cell stemness, proliferation, and lenvatinib resistance. Furthermore, our data demonstrate that USP15 enhances LGALS3 stability and maintains its upregulated expression through deubiquitination modification, potentially revealing the mechanism of action of LGALS3 in HCC(Supplementary Fig. S3).

The PI3K/AKT/mTOR pathway is a classical pathway that responds to insulin signaling and acts downstream of tyrosine kinase receptor to function in cell growth, migration, and survival. In HCC, approximately 5%–10% of patients have abnormal activation of the PI3K/AKT/mTOR pathway, and inhibiting this pathway can significantly inhibit HCC cell proliferation and invasion [28]. Moreover, previous studies have shown that USP15 promotes colon cancer proliferation and invasion via the AKT/mTOR pathway [29]. In our study, we found that USP15 can interact with LGALS3 and activate the AKT/mTOR pathway by phosphorylating the AKT and mTOR proteins to exert cancer-promoting functions. However, the mechanism by which USP15 activates AKT/mTOR signaling remains unclear. TGF-β has been reported to promote *USP15* mRNA translation through PI3K/AKT signaling, thereby activating the AKT/mTOR pathway [30].

RNA modification is a dynamic and reversible process, with m6A modification the most common of all RNA epigenetic alterations discovered to date. It regulates RNA transcription, editing, translation, and stability, playing a leading role in tumor cell proliferation and invasion [31]. The up-regulation or down-regulation of m6A modification and its regulatory factors are critical in HCC occurrence and development [32]. Mettl3 is upregulated in various HCC cell lines, resulting in increased m6A modification and promoting HCC progression. However, the mechanism of action of Mettl3 in the context of HCC remains unclear [22]. Our study demonstrated that USP15 has numerous Mettl3 binding sites and that Mettl3 is highly expressed in HCC. Moreover, Mettl3 expression level directly influenced *USP15* m6A enrichment, indicating that USP15 expression upregulation in HCC is likely mediated by m6A modification. Subsequently, we found that Mettl3 expression level can affect *USP15* mRNA stability. Therefore, we conclude that USP15 upregulation in HCC is likely mediated through m6A modification.

Despite efforts to ensure a rigorous experimental design, our study had some limitations. Although we found that USP15 increased the resistance to lenvatinib in HCC, we did not study specimens from patients with HCC treated with lenvatinib, making it impossible for us to objectively evaluate whether USP15 can be used as a marker for targeted treatment resistance to lenvatinib in the clinic. In addition, the specific mechanism underlying USP15-mediated activation of the AKT/mTOR pathway was not verified, and will be investigated in subsequent studies.

## Materials and methods

### Patients and clinical specimens

Tumor and corresponding normal control tissue specimens were collected from 52 patients diagnosed with HCC who underwent liver resection at the Second Hospital of Jilin University from January 2019 to January 2021. Patient data were also collected, including sex, age, etiology, serum AFP, liver cirrhosis, tumor stage, tumor size, smoking history, alcohol consumption history, and microvascular invasion. Cancer staging was based on the HCC-TNM staging of the International Union Against Cancer. All human sample studies were reviewed and approved by the Ethics Committee of the Second Hospital of Jilin University, and informed consent was obtained from all patients. All procedures were in accordance with the Declaration of Helsinki.

### Cell culture

The human liver immortalized cell line, LO2, and the HCC cell lines, Hep3B, SK-Hep1, Huh7, HCC-LM3, and SNU-449, were purchased from the American Type Bioresource Collection. Cells were cultured in appropriate complete medium (DMEM; BBI) containing 10% fetal bovine serum (BBI), 1% penicillin (100 U/ml), and streptomycin (100 U/ml). All cells were cultured in a humidified incubator at 37°C with 5% CO_2_.

### Reagents and antibodies

Trypsin (0.25%), TRIzol, and RIPA lysis buffer were purchased from Sangon Biotech (Shanghai, China). Lipofectamine 3000 was from Invitrogen (Grand Island, NY, USA). An RNA Reverse Transcription Kit was purchased from Thermo (Waltham, Massachusetts, USA). Antibodies (anti-USP15, anti-LGALS3, anti-Mettl3, anti-AKT, anti-p-AKT, anti-mTOR, anti-p-mTOR, and anti-GAPDH) were all purchased from Proteintech (Rosemont, Illinois. USA). Unless otherwise stated, all other chemical reagents were purchased from Sigma-Aldrich (St. Louis, Missouri, USA).

### Quantitative real-time PCR (qRT-PCR)

Total RNA was extracted from patient tissue or cell lines using TRIzol, according to the manufacturer’s instructions, and reverse transcription was performed using an RNA reverse transcription kit. Real-time qRT-PCR analysis was performed using PowerUp SYBR Green premix (Thermo, USA), according to the manufacturer’s instructions, with three replicate wells for each sample, reaction data were exported, and the mRNA expression of the target gene calculated according to the detected Ct value as 2^−ΔΔCt^. Primer sequences used in this study are shown in Supplementary Table S1.

### Western blot (WB)

Tissues or cell pellets were homogenized in RIPA buffer. Protein concentration was determined using a BCA protein assay kit (ThermoScience, Shanghai, China). Equal amounts of protein were separated by SDS-PAGE and then transferred to polyvinylidene difluoride membranes (Bio-Rad, Shanghai, China). Membranes were blocked with 5% skim milk for 1 h at room temperature, then primary antibodies were added and incubated overnight at 4 °C in a refrigerator. The membranes were washed three times with TBST buffer solution for 10 min each, secondary antibodies added and incubated at room temperature for 1 h, then discarded, and washed three times with TBST buffer solution for 10 min each. After adding ultra-sensitive ECL chemiluminescent working solution (avoiding bubbles), blots were imaged using a chemiluminescent system, photographed, and analyzed.The full gel and blot images for this study are provided in an additional file (please refer to Additional File 1).

### Mass spectrometry

Successfully transfected HCC cells were immunoprecipitated using anti-DYKDDDDK magnetic agarose (ThermoScience, Shanghai, China), washed five times with PBS for 10 min each, then resuspended in 2× SDS loading buffer, and boiled for 10 min in a water bath before gel electrophoresis. Mass spectrometry analysis was performed after staining with Coomassie brilliant blue.

### Ubiquitination assay

Successfully transfected HCC cells were treated with MG132 for 4 h, then NP-40 lysis buffer was added to lyse the cells. Samples were incubated overnight at 4°C with an HA tag antibody, then Protein A/G PLUS-Agarose was added. After 6 h, the immune complex was collected and centrifuged at 804 RCF for 5 min at 4 °C. The precipitates were collected and analyzed by WB.

### Protein half-life assay

HCC cells were treated with 20 μg/ml cycloheximide to block protein synthesis. Proteins were extracted after 0, 4, 8, 12, and 16 h, and the expression of related proteins was analyzed by WB.

### MeRIP-qPCR

HCC cells were treated with TRIzol to extract intracellular RNA. Anti-m6A or IgG was bound to protein A/G magnetic beads. Then, 100 μg of previously extracted RNA was mixed with the prepared protein A/G magnetic beads and eluted twice with N6-methyladenosine 5’-monophosphate sodium salt at 4 °C for 1 h each time, followed by qRT-PCR.

### Actinomycin D assay

Each group of cells was treated with actinomycin D to inhibit transcription. Subsequently, RNA was extracted from each group and tested by qRT-PCR.

### Tumor formation in nude mice

Huh7 HCC cells with USP15 knocked down (Huh7-shUSP15) and wild-type Huh7 cells were used for these experiments. BALB/c-nu nude mice (*n* = 20, 5 weeks old, male) were purchased from Weitonglihua Laboratory Animal Technology Co., Ltd. (Beijing, China) and randomly divided into four groups (*n* = 5 mice per group), to investigate whether USP15 promotes HCC progression and whether USP15 can enhance HCC resistance to lenvatinib. No blinding methods were employed in this experiment. Both cell lines were suspended in PBS and inoculated subcutaneously into nude mice using a fine needle. Once tumor volume reached 0.5 cm^3^, the treatment groups received daily injections of lenvatinib (50 mg/kg) via the tail vein. After 25 days, mice were euthanized and the tumors were excised. Tumor size was measured with a vernier caliper, and tumor volume was calculated. For further immunohistochemistry, tumors were embedded in paraffin. All animal experiments were performed in accordance with the official guidelines of the Animal Ethics Committee.

### Other assays

Details on other experimental methods, including immunohistochemistry, transfection, co-immunoprecipitation, cell growth assay, colony formation assay, and spheroid formation assay are provided in Additional File 2.

### Statistical analysis

All statistical data are expressed as mean ± standard deviation. Statistical analysis was performed using GraphPad Prism v8.0 (GraphPad, Inc., USA) and Statistical Package for Social Sciences v22.0 (SPSS, Inc., Chicago, IL, USA). *P* < 0.05 was considered statistically significant.

## Supplementary information

qRT-PCR and WB detection of differences in USP15 expression between the immortalized human liver cell line and the HCC cell lines

Lentivirus transfection to construct a Hep-3B cell line stably overexpressing USP15 and Huh-7 and HCC-LM3 cell lines with USP15 knocked down

Schematic diagram illustrating the role and mechanism of USP15

Primer sequences in the study

Relationship between USP15 and clinicopathological parameters in 52 HCC patients

The full gel and blot images in this study

Details on other experimental methods

## Data Availability

The datasets used and/or analyzed during the current study are available from the corresponding author on reasonable request.
