# Supplementary figures and images for: N6-methyladenosine-modification of USP15 regulates chemotherapy resistance by inhibiting LGALS3 ubiquitin-mediated degradation via AKT/mTOR signaling activation pathway in hepatocellular carcinoma
Source: Cell Death Discov. 2025 Jan 10;11:3. doi: 10.1038/s41420-024-02282-y (PMC11724082; doi:10.1038/s41420-024-02282-y)

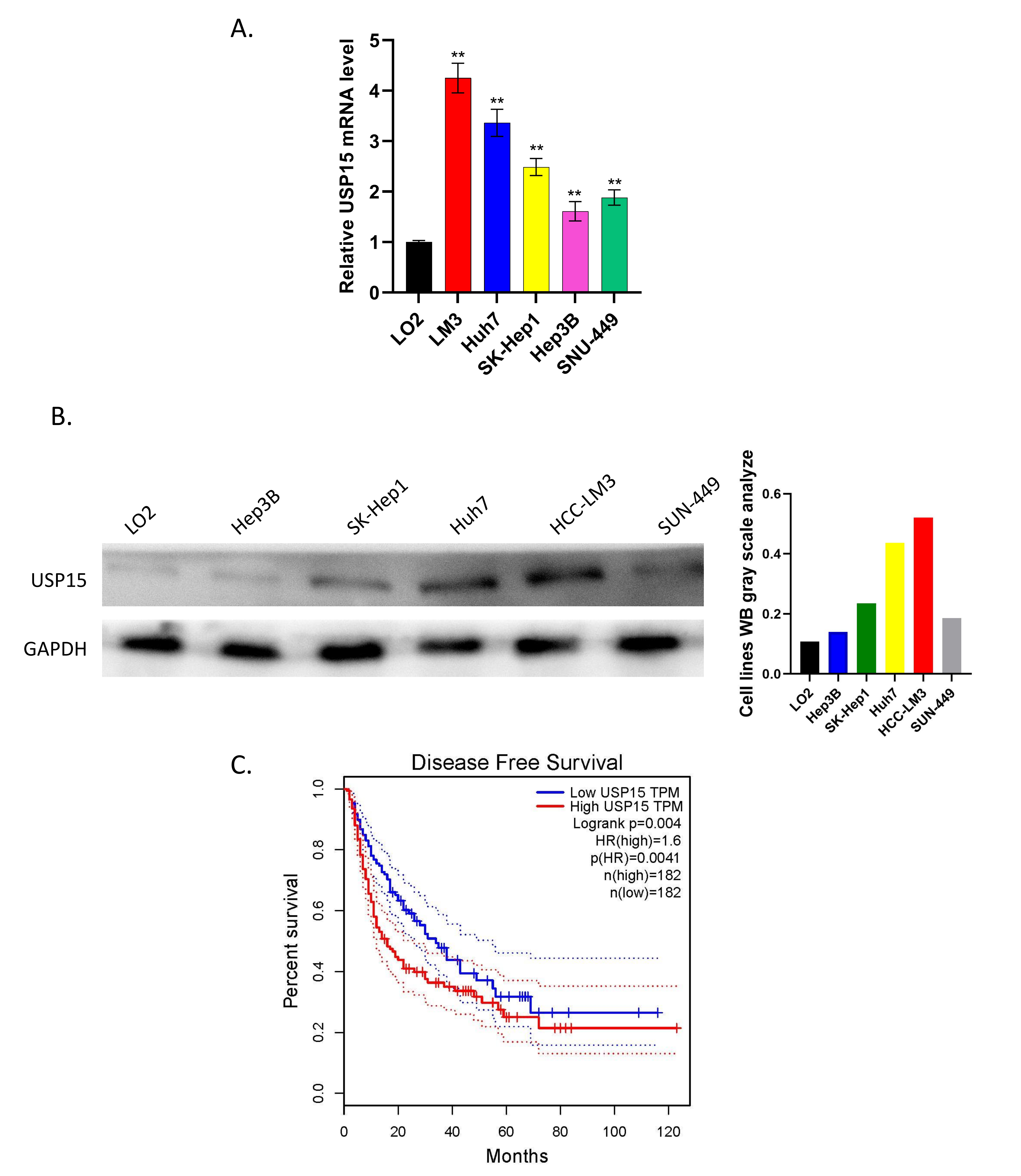

Supplement: Supplementary file 1 — qRT-PCR and WB detection of differences in USP15 expression between the immortalized human liver cell line and the HCC cell lines [file 41420_2024_2282_MOESM1_ESM.png]

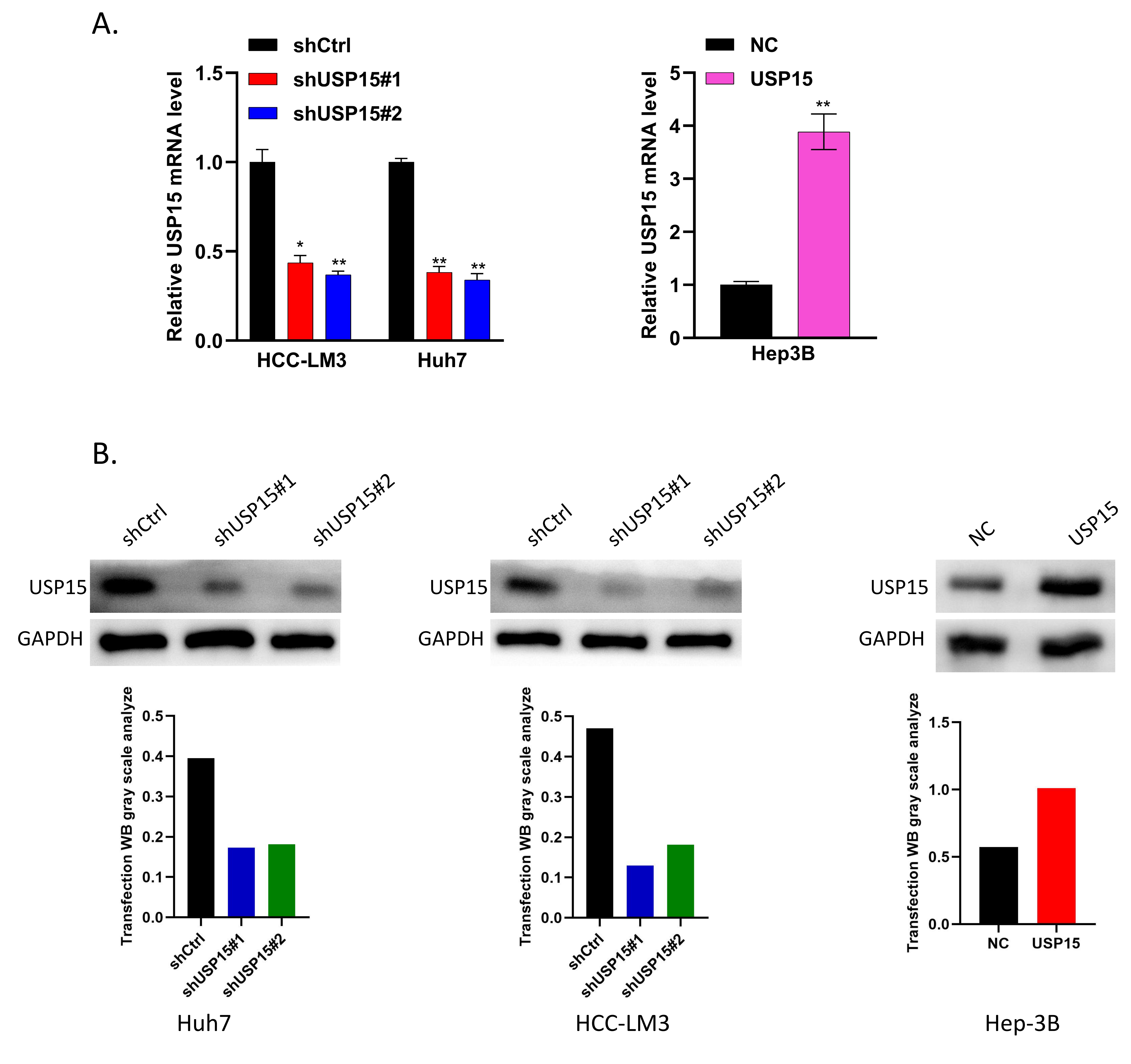

Supplement: Supplementary file 2 — Lentivirus transfection to construct a Hep-3B cell line stably overexpressing USP15 and Huh-7 and HCC-LM3 cell lines with USP15 knocked down [file 41420_2024_2282_MOESM2_ESM.png]

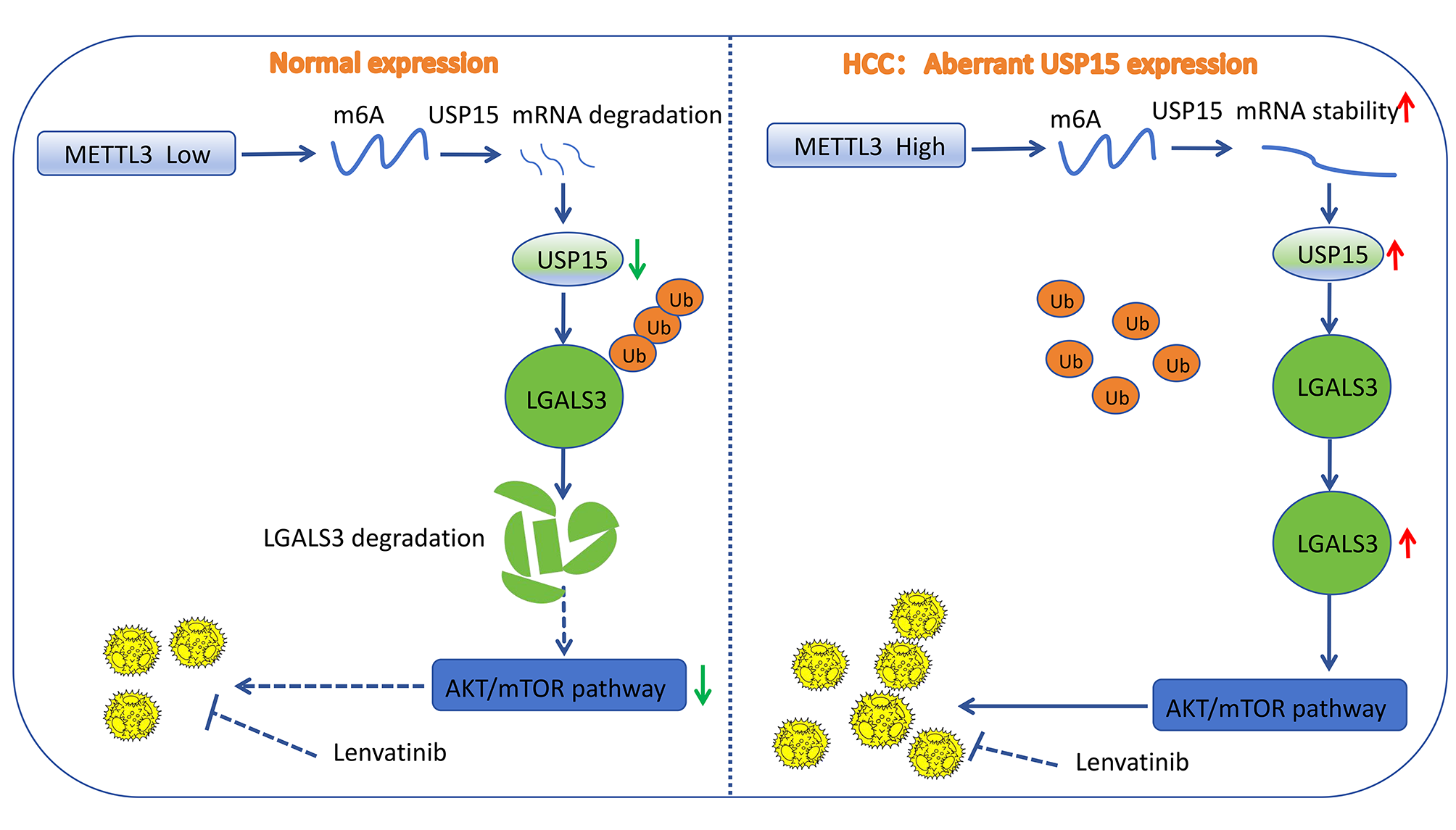

Supplement: Supplementary file 3 — Schematic diagram illustrating the role and mechanism of USP15 [file 41420_2024_2282_MOESM3_ESM.png]
