# Supplementary material for: N6-methyladenosine-modification of USP15 regulates chemotherapy resistance by inhibiting LGALS3 ubiquitin-mediated degradation via AKT/mTOR signaling activation pathway in hepatocellular carcinoma
Source: Cell Death Discov. 2025 Jan 10;11:3. doi: 10.1038/s41420-024-02282-y (PMC11724082; doi:10.1038/s41420-024-02282-y)
Supplement: Supplementary file 6 — The full gel and blot images in this study [file 41420_2024_2282_MOESM6_ESM.ppt]

## Slide 1
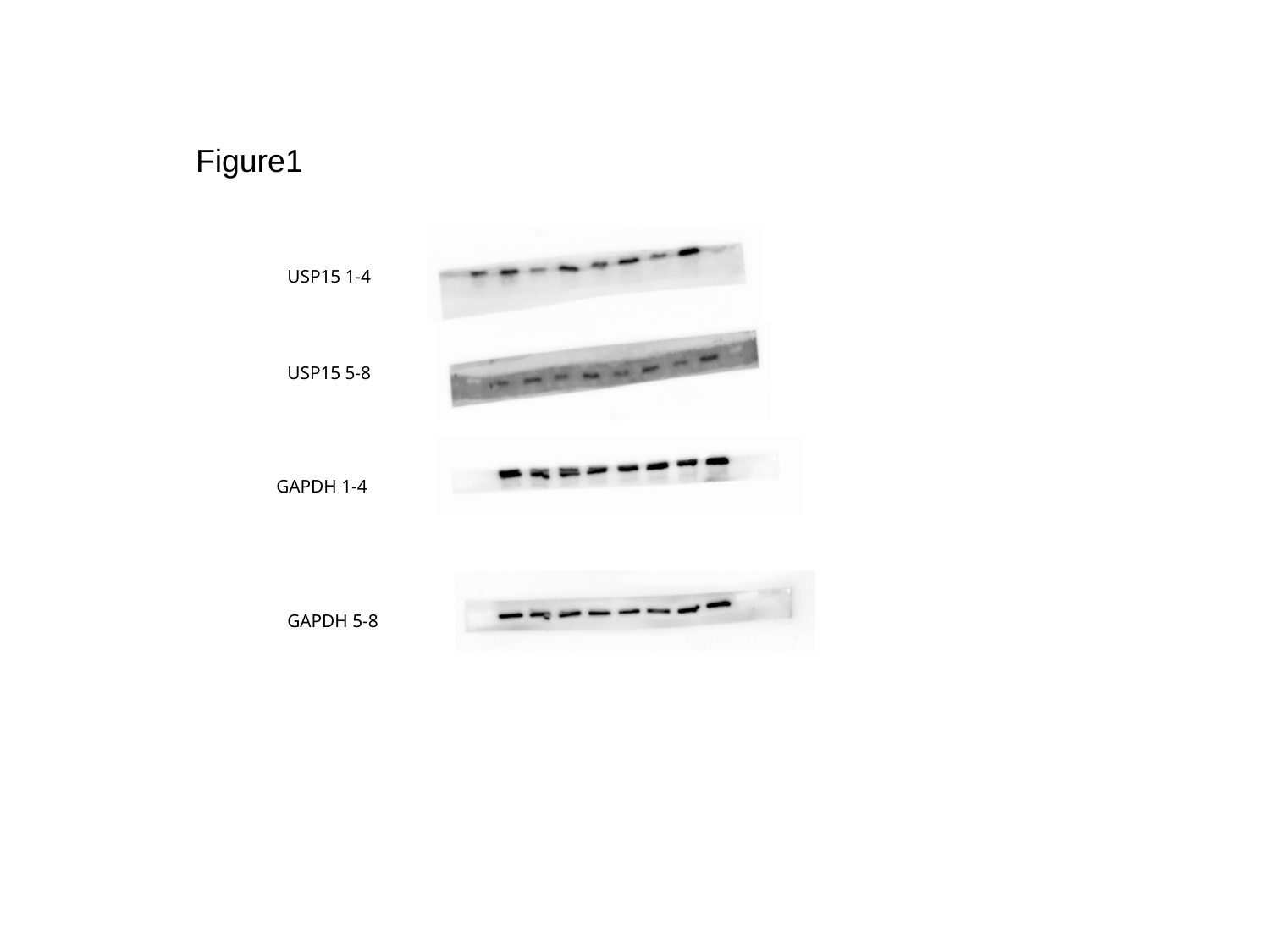

Figure1
USP15 1-4
USP15 5-8
GAPDH 1-4
GAPDH 5-8

## Slide 2
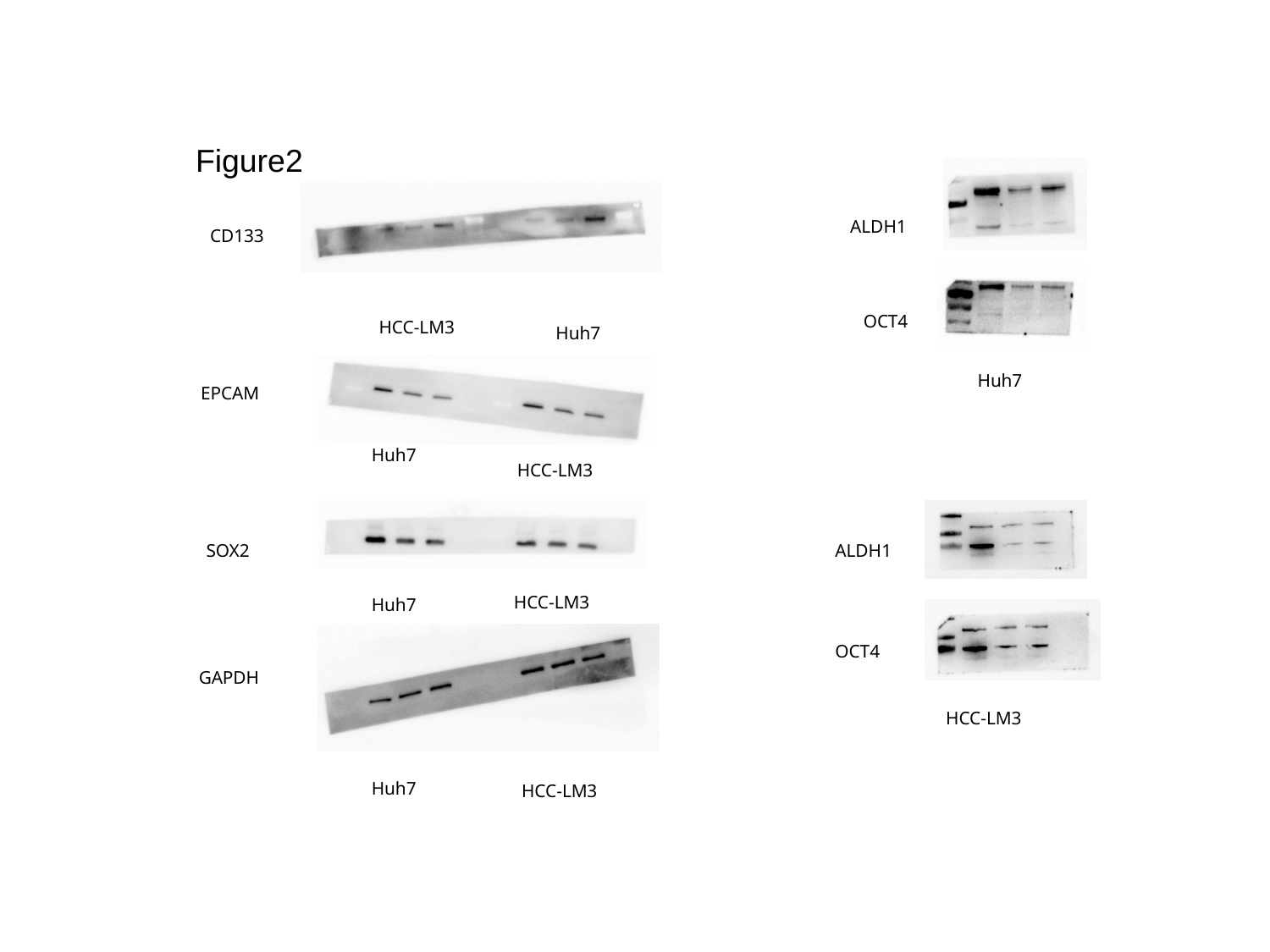

Figure2
ALDH1
CD133
EPCAM
SOX2
GAPDH
OCT4
HCC-LM3
Huh7
Huh7
Huh7
HCC-LM3
ALDH1
HCC-LM3
Huh7
OCT4
HCC-LM3
Huh7
HCC-LM3

## Slide 3
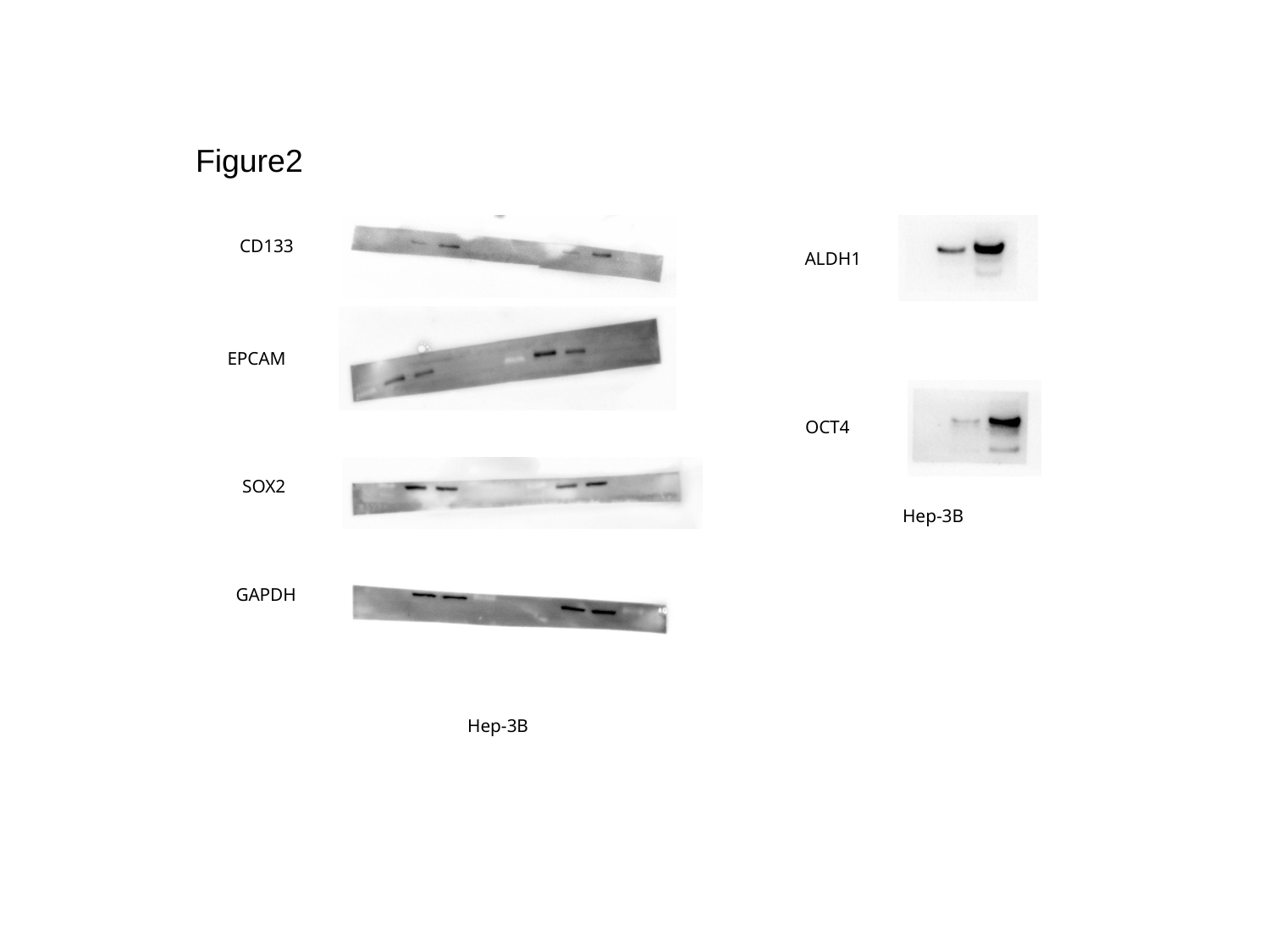

Figure2
CD133
EPCAM
SOX2
GAPDH
Hep-3B
ALDH1
OCT4
Hep-3B

## Slide 4
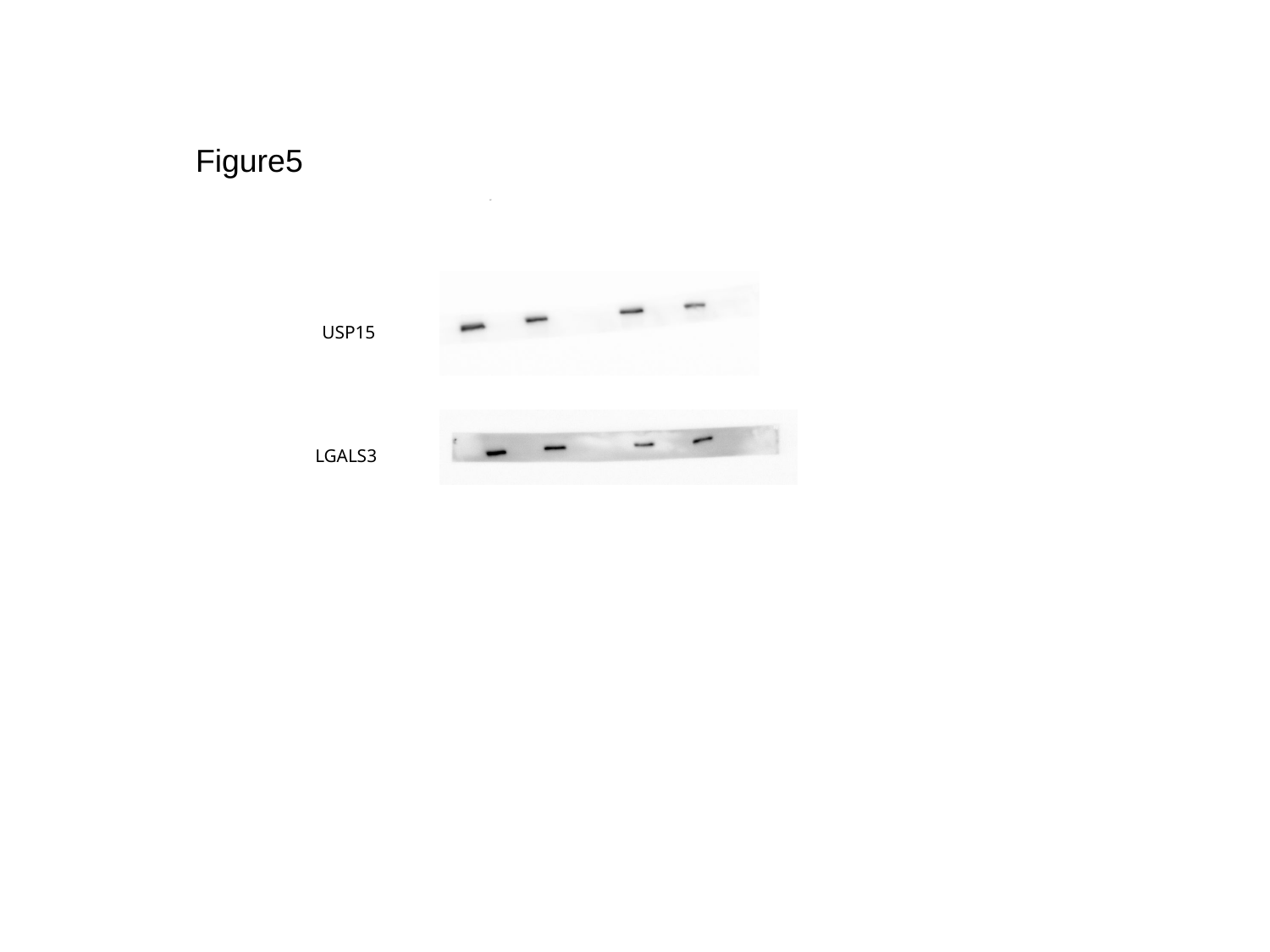

Figure5
IP
USP15
LGALS3

## Slide 5
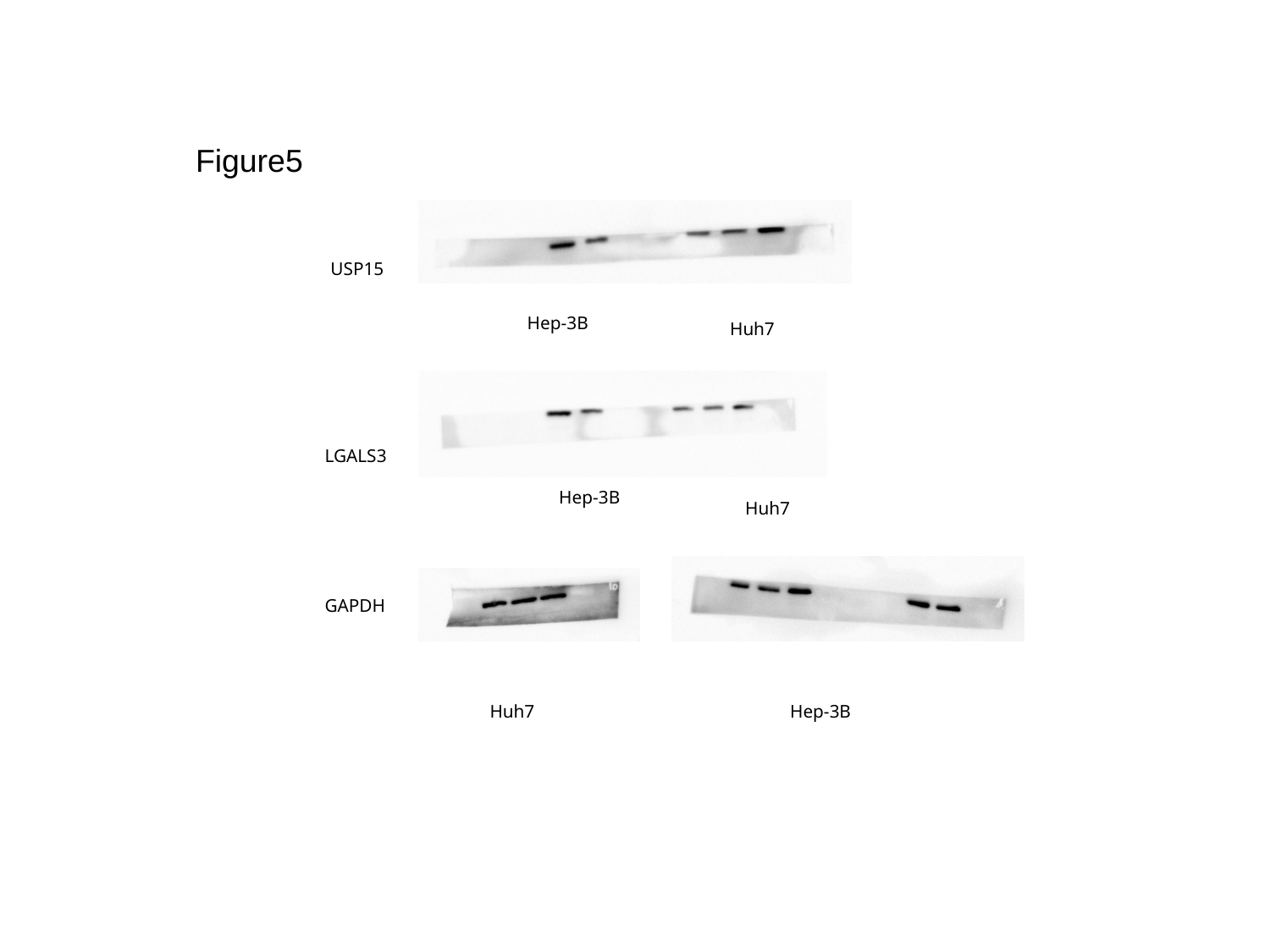

Figure5
USP15
Hep-3B
Huh7
LGALS3
Hep-3B
Huh7
GAPDH
Huh7
Hep-3B

## Slide 6
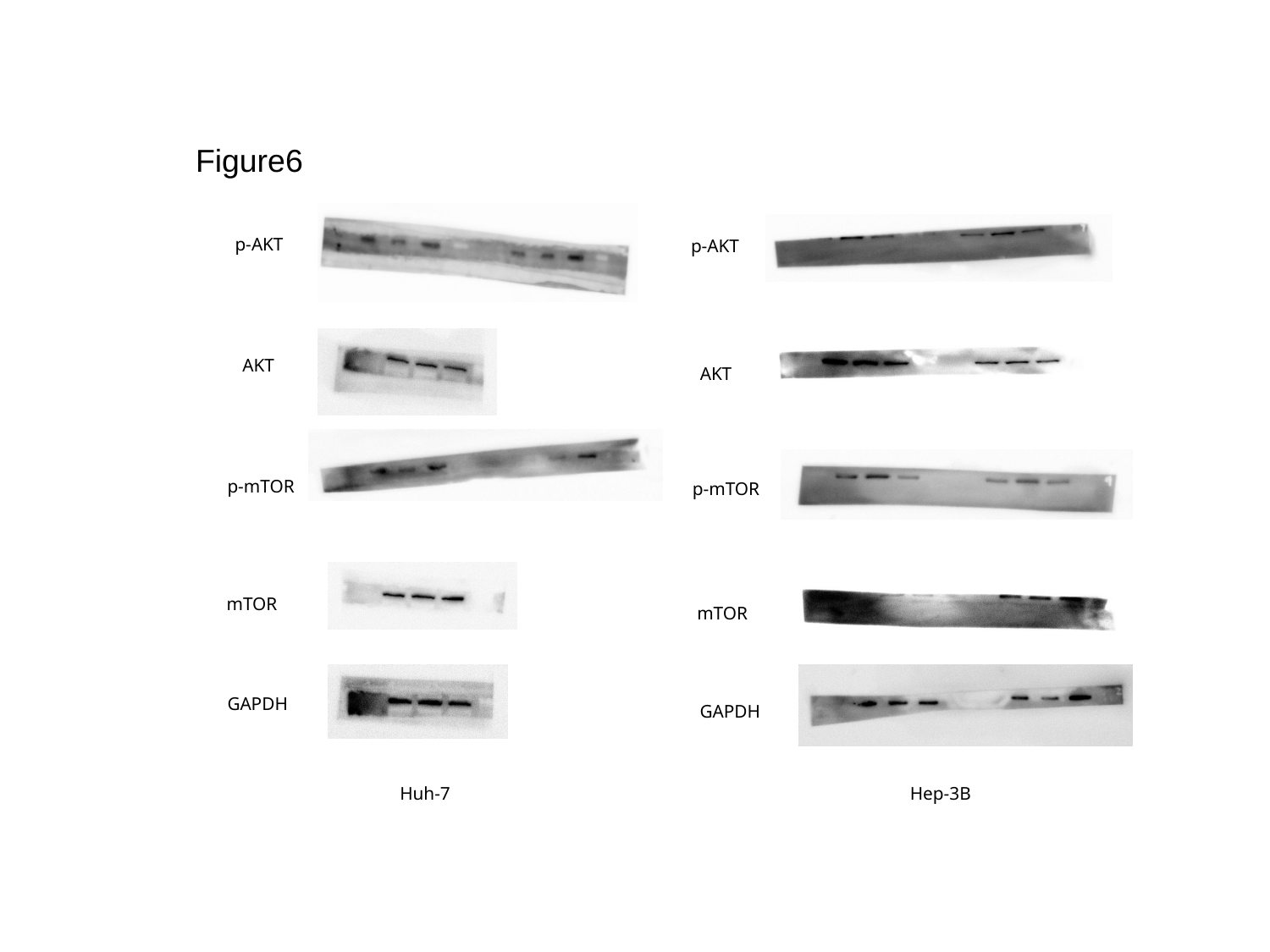

Figure6
p-AKT
p-AKT
AKT
AKT
p-mTOR
p-mTOR
mTOR
mTOR
GAPDH
GAPDH
Huh-7
Hep-3B

## Slide 7
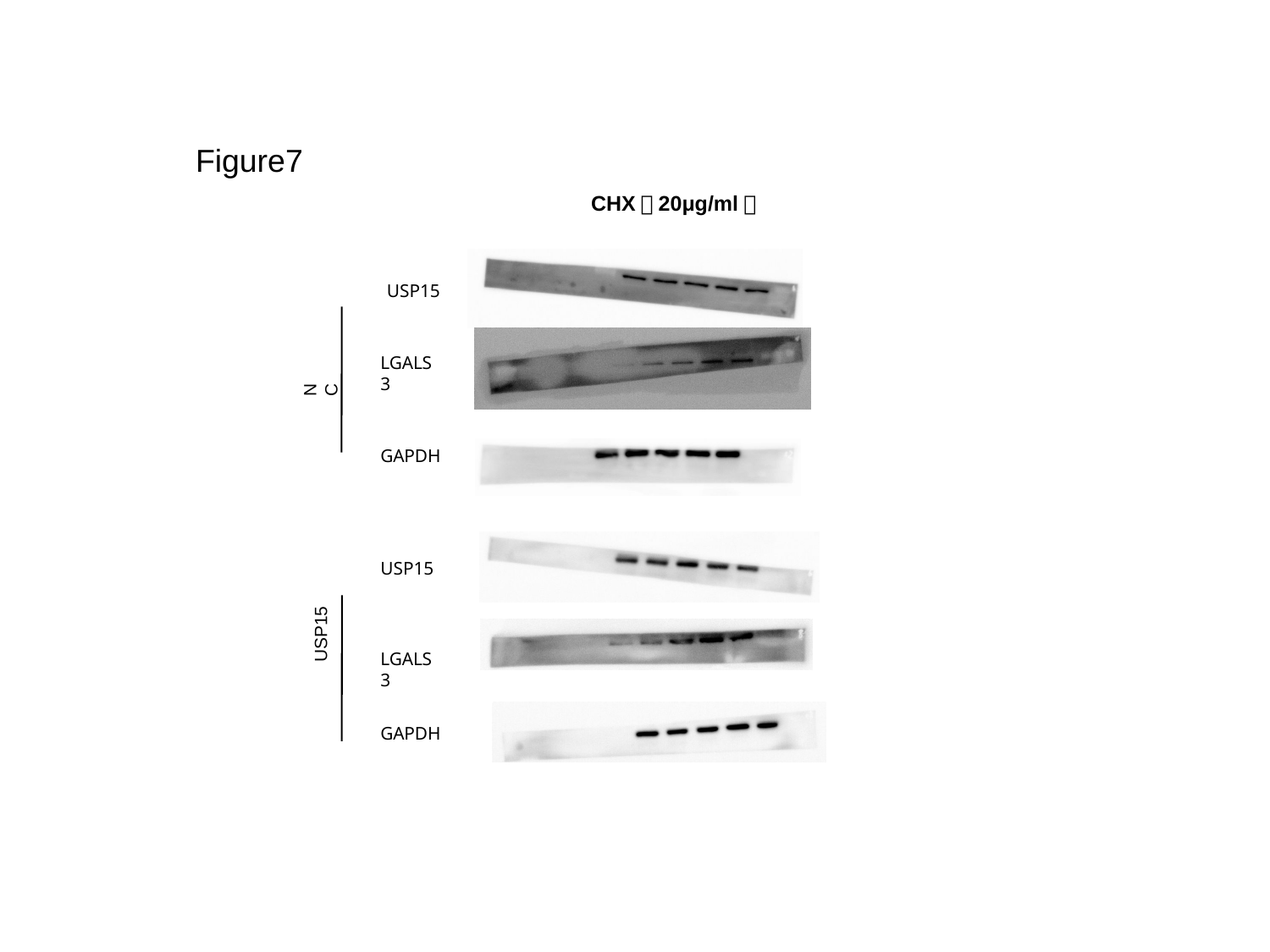

Figure7
CHX（20μg/ml）
USP15
LGALS3
NC
GAPDH
USP15
USP15
LGALS3
GAPDH

## Slide 8
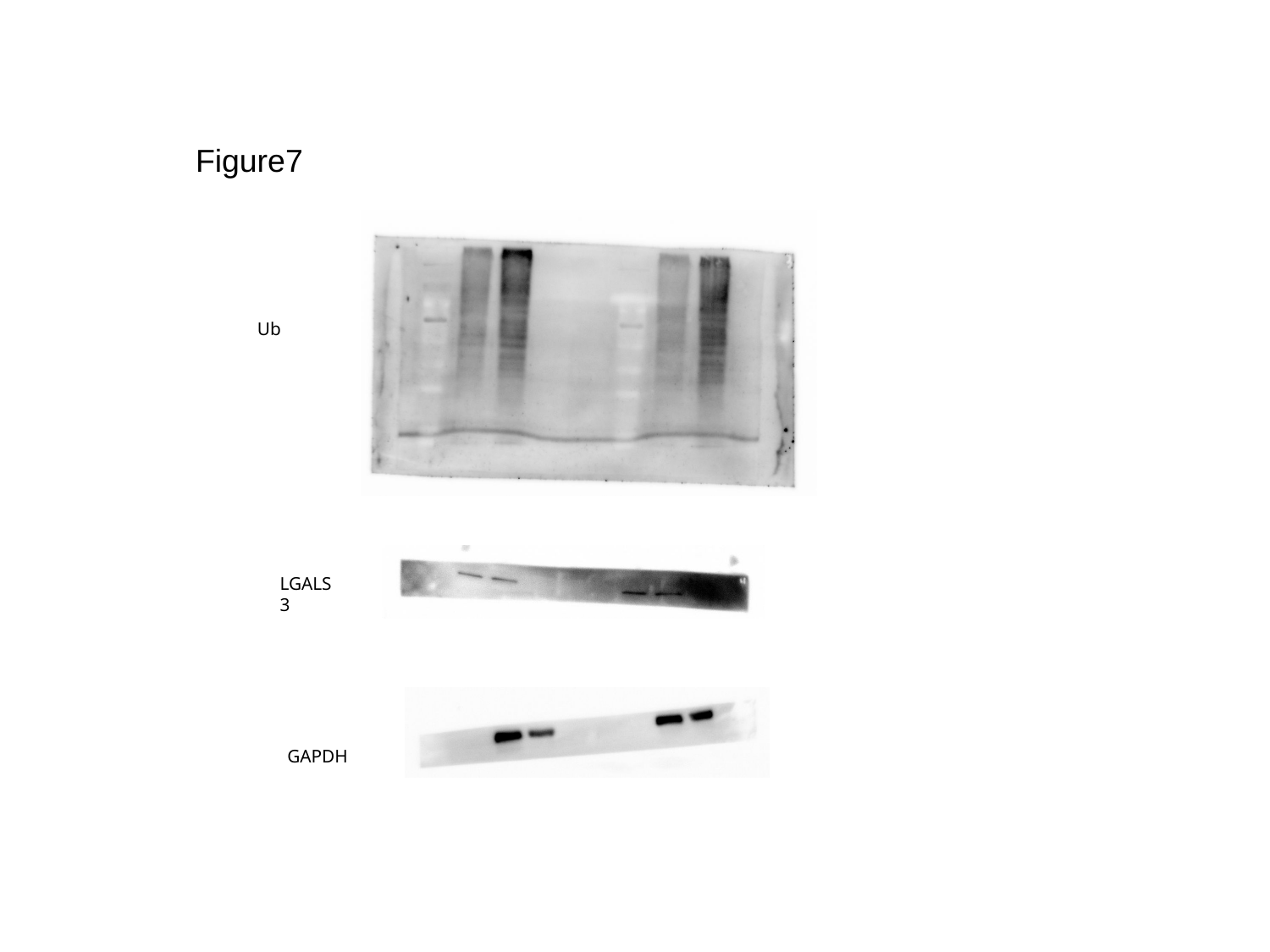

Figure7
Ub
LGALS3
GAPDH

## Slide 9
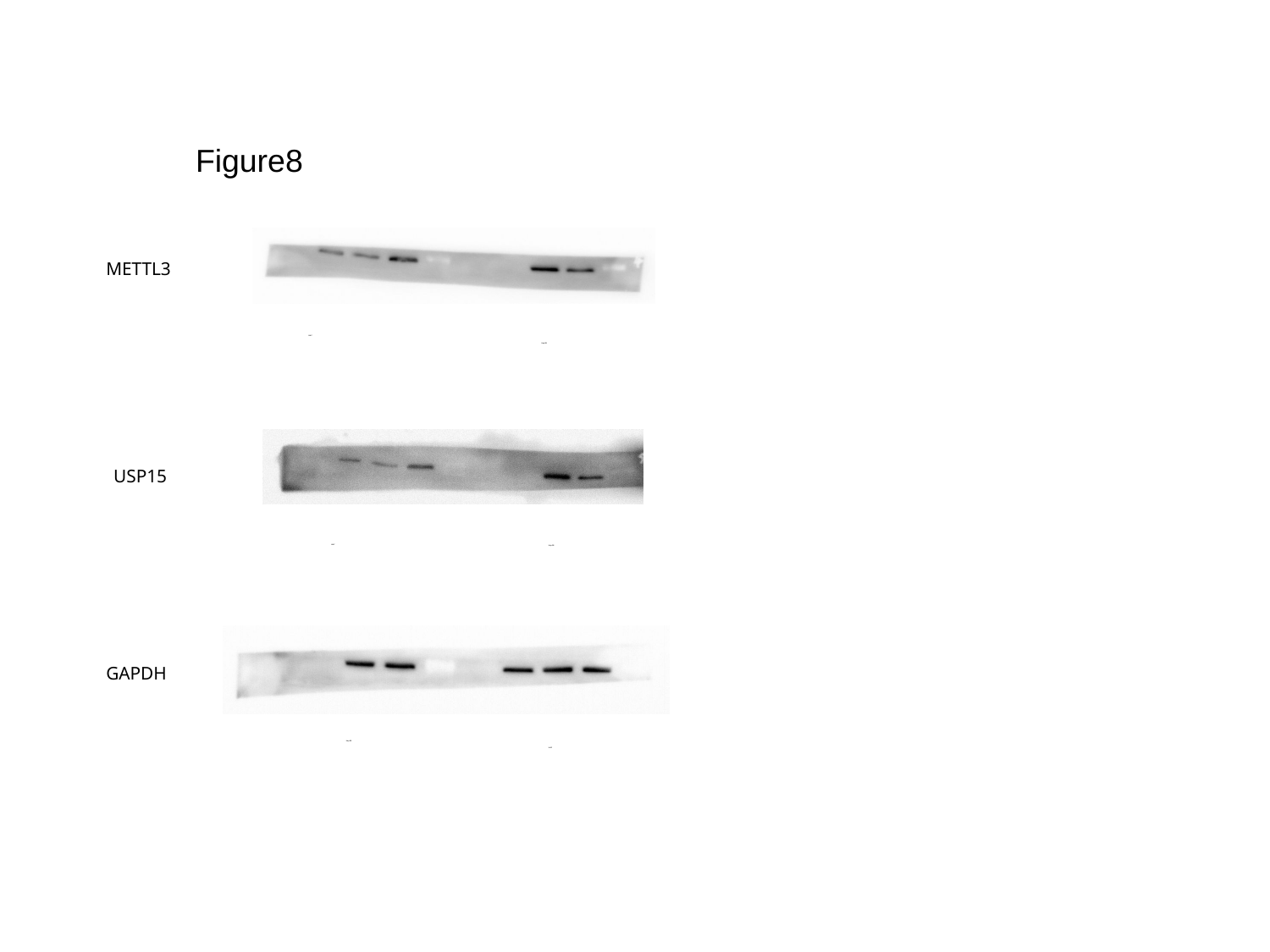

Figure8
METTL3
Huh7
Hep-3B
USP15
Huh7
Hep-3B
GAPDH
Hep-3B
Huh7

## Slide 10
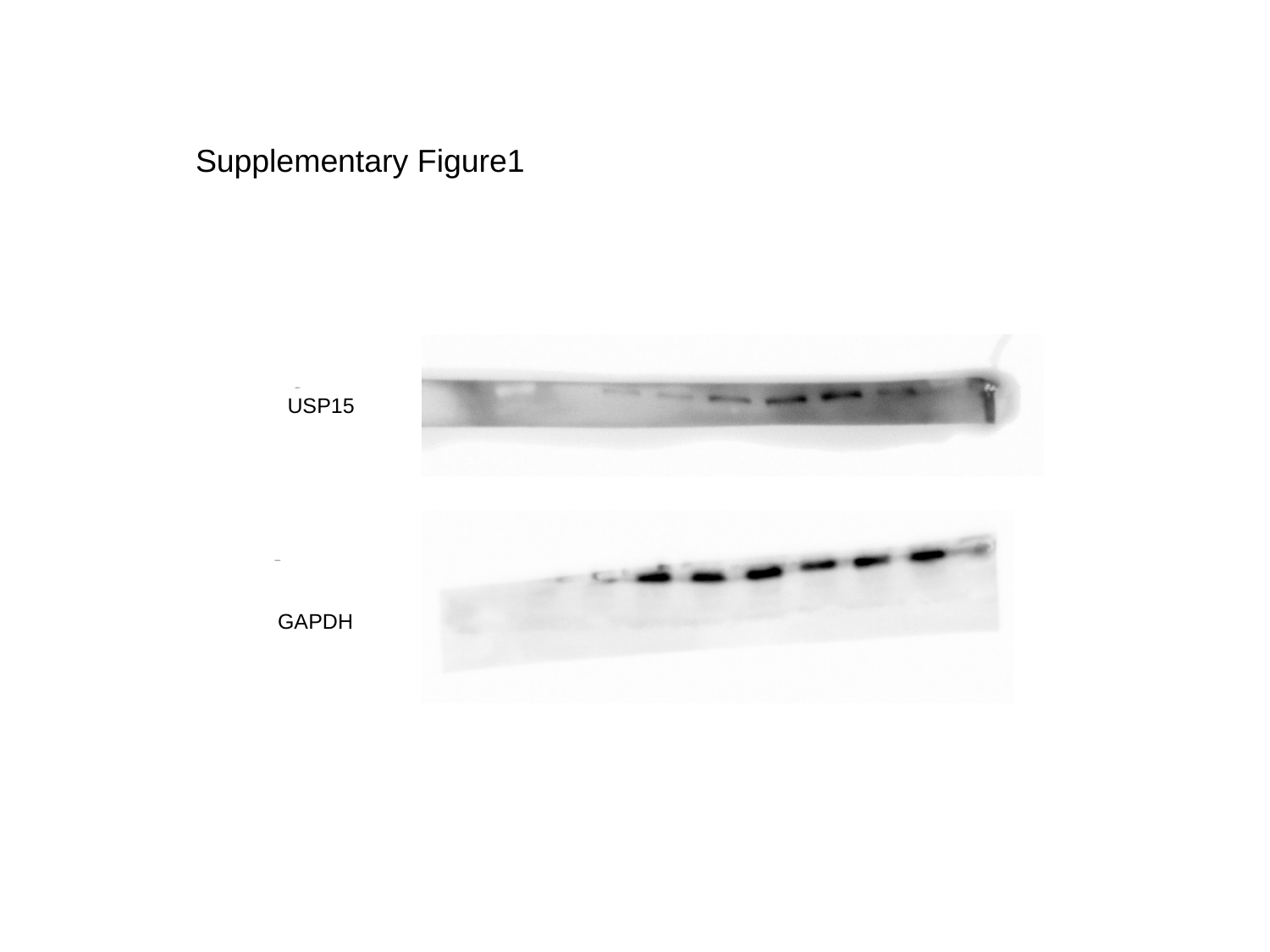

Supplementary Figure1
USP15
GAPDH
USP15
GAPDH

## Slide 11
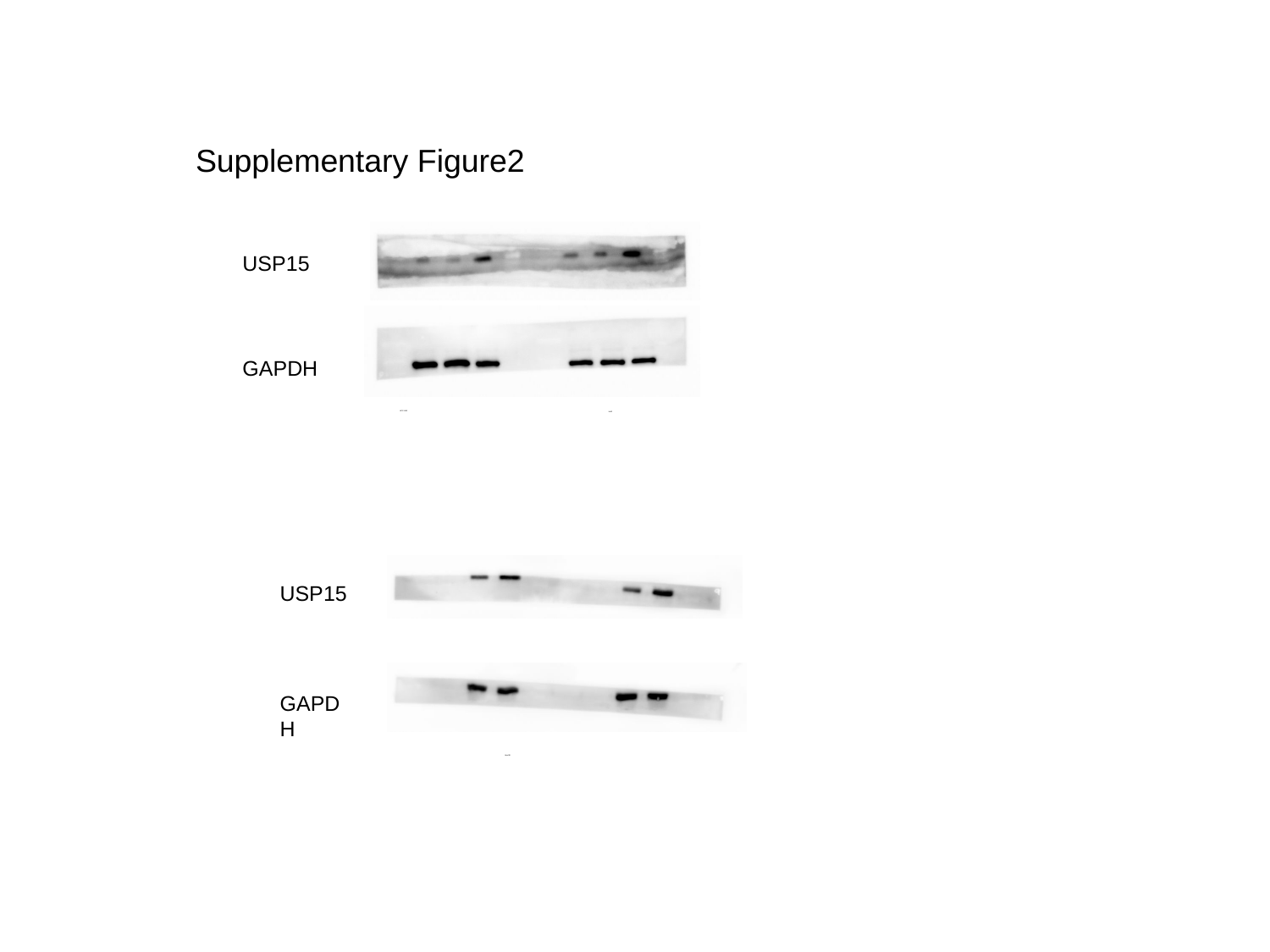

Supplementary Figure2
USP15
GAPDH
HCC-LM3
Huh7
USP15
GAPDH
Hep-3B
