# Supplementary material for: N6-methyladenosine-modification of USP15 regulates chemotherapy resistance by inhibiting LGALS3 ubiquitin-mediated degradation via AKT/mTOR signaling activation pathway in hepatocellular carcinoma
Source: Cell Death Discov. 2025 Jan 10;11:3. doi: 10.1038/s41420-024-02282-y (PMC11724082; doi:10.1038/s41420-024-02282-y)
Supplement: Supplementary file 7 — Details on other experimental methods [file 41420_2024_2282_MOESM7_ESM.docx]

Immunohistochemistry

Paraffin-embedded tissue sections were provided by the Department of Pathology, Second Hospital of Jilin University. Paraffin sections were dewaxed in xylene and then hydrated with graded ethanol. Antigen retrieval was performed by immersing sections in citrate buffer (0.01 M, pH 6.0), after which endogenous peroxidase activity was blocked with 3% hydrogen peroxide at room temperature for 15 min. Paraffin sections were blocked with 5% goat serum for 1 h and incubated with anti-USP15 (1:100) overnight at 4°C. Sections were then washed three times with PBS for 10 min each, incubated with secondary antibodies at room temperature for 1 h, and washed again with PBS for 10 min each. A DAB kit (ThermoScience, Shanghai, China) was used for color development, and USP15 expression in tissues was observed under a microscope after re-staining with hematoxylin.

Plasmid, lentivirus, and siRNA transfection

Plasmids, lentivirus, and siRNA were purchased from GenePharma (Shanghai, China). Prepared virus solution was added to HCC cells and transfected using Lipofectamine3000 transfection reagent; blank vector was also transfected. Cells were harvested 72 h after transfection, and 5 μg/ml puromycin used to select successfully transfected HCC cells. Target gene expression was verified by qRT/PCR and WB.

Co-immunoprecipitation (Co-IP)

Successfully transfected HCC cells were lysed using NP-40 lysis buffer (ThermoScience, Shanghai, China), and primary antibody and immunoglobulin (IgG; Cell Signaling Technology) added to the cell lysate, shaken slowly on a shaker, and incubated overnight. Protein A/G PLUS-Agarose (ABcam, Shanghai, China) was then added according to the manufacturer's instructions, and incubated on a shaker overnight. All the above operations were performed at 4°C. After collection, samples were washed three times with pre-cooled PBS for 10 min each time, and then added to 2× SDS loading buffer, boiled in a heated water bath for 5 min, and then subjected to SDS-PAGE and WB.

Cell growth assay

HCC cells were seeded into 96-well plates at 1 × 103 cells per well, cell counting reagent (CCK8) added into the wells, and absorbance at 450 nm measured to assess cell viability.

Colony formation assay

HCC cells were seeded into 6-well plates at 500 cells per well, before addition of 1.57 μM lenvatinib or DMSO (control) and culture for 2 weeks. Cells were fixed with methanol for 10 min, then stained with crystal violet for 15 min, and cell colonies observed and counted under a microscope.

Spheroid formation assay

HCC cells were seeded into 6-well plates at 500 cells per well, and cultured in medium containing B27, EGF, and bFGF for 7 days. Sphere formation was observed under a microscope. Cells were re-digested, centrifuged, counted, and re-seeded into 6-well plates at 500 cells per well. The previous operation was repeated, and sphere formation observed again after re-culture for 7 days.
